# Discovery of New Carbonyl Reductases Using Functional Metagenomics and Applications in Biocatalysis

**DOI:** 10.1002/adsc.202100199

**Published:** 2021-05-04

**Authors:** Sophie A. Newgas, Jack W. E. Jeffries, Thomas S. Moody, John M. Ward, Helen C. Hailes

**Affiliations:** ^1^ Department of Chemistry University College London 20 Gordon Street London WC1H 0AJ U.K.; ^2^ Department of Biochemical Engineering Bernard Katz Building University College London London WC1E 6BT U.K.; ^3^ Almac Sciences Department of Biocatalysis and Isotope Chemistry Almac House, 20 Seagoe Industrial Estate Craigavon BT63 5QD Northern Ireland U.K.; ^4^ Arran Chemical Company Unit1 Monksland Industrial Estate Athlone N37 DN24 Co. Roscommon Ireland.

**Keywords:** biocatalysis, carbonyl reductases, short-chain reductases, Wieland-Meischer ketone, functional metagenomics

## Abstract

Enzyme discovery for use in the manufacture of chemicals, requiring high stereoselectivities, continues to be an important avenue of research. Here, a sequence directed metagenomics approach is described to identify short chain carbonyl reductases. PCR from a metagenomic template generated 37 enzymes, with an average 25% sequence identity, twelve of which showed interesting activities in initial screens. Six of the most productive enzymes were then tested against a panel of 21 substrates, including bulkier substrates that have been noted as challenging in biocatalytic reductions. Two enzymes were selected for further studies with the Wieland Miescher ketone. Notably, enzyme SDR‐17, when co‐expressed with a co‐factor recycling system produced the *anti*‐(4a*R*,5*S*) isomer in excellent isolated yields of 89% and 99% *e.e*. These results demonstrate the viability of a sequence directed metagenomics approach for the identification of multiple homologous sequences with low similarity, that can yield highly stereoselective enzymes with applicability in industrial biocatalysis.

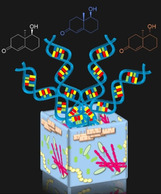

## Introduction

In recent years the use of biocatalysts has continued to grow and play an important role in the fine chemical, pharmaceutical, food, biofuel and waste industries. Compared to traditional organic chemistry strategies, biocatalysts typically use mild reaction conditions, do not require toxic transition metals, organic solvents or extreme temperatures and can achieve excellent chemo‐ and stereoselectivities. Biocatalysts are now recognised industrially to provide a sustainable alternative to conventional chemical catalysts.^[1]^ It is for these reasons that the market in industrial enzyme use is predicted to grow to £10 billion by 2024.^[2]^

Carbonyl reductases, ketoreductases, and alcohol dehydrogenases (ADHs) (EC 1.1.1) cover a large group of enzymes that reduce a ketone or an aldehyde group to an alcohol, or perform the reverse reaction, using either NADH or NADPH as a cofactor. One class of ADHs known as short chain dehydrogenases/reductases (SDRs) form a diverse family of proteins, which all display similar α/β folding patterns with a central β‐sheet, typical of the Rossmann‐fold.^[3]^ With oxidation and reduction accounting for the second largest number of studies on biocatalysis, SDRs are an important area of focus.^[4]^ More importantly SDRs also provide an efficient route to single enantiomer alcohols.^[2]^ In the pharmaceutical industry the production of single isomer drugs is becoming ever more important, in 2015 all chiral drugs approved for sale by the FDA bar one, were single enantiomers.[^5^, ^6^]

One of the limitations of using SDRs is a lack of enzymes that can accept large lipophilic substrates or carbonyl moieties flanked by sterically challenging groups, as well as the use of the expensive cofactor NAD(P)H. Most SDRs preferentially generate the (*S*)‐alcohol in line with Prelog's rule and the limited number of anti‐Prelog enzymes, which will generate the (*R*)‐alcohol, is another stumbling block.[^7^, ^8^] In addition, non‐engineered biocatalysts can suffer from low organic solvent tolerances, narrow working pH ranges, and low thermostabilities. The discovery of new enzymes is a key strategy to overcome these issues. Identifying enzymes from metagenomic sources, where DNA is extracted from an environmental sample and analysed using high throughput sequencing technologies, has proved to be a valuable method. With the increasing availability and the decreasing cost of sequencing methods, there has been a rise in studies using functional metagenomics.[^9^, ^10^, ^11^]

In recent work, we have utilised this approach by collecting metagenomic material from the human oral cavity and a domestic drain, sequencing the DNA and assembling the shorter reads into larger contiguous stretches of DNA (contigs), creating an *in silico* library. Using this library, open reading frames, operons and enzymes can be identified and this approach has been used to discover new transketolases, ene‐reductases and transaminases.[^9^, ^10^, ^12^, ^13^] After using PCR to amplify identified DNA, the enzymes were cloned, and assayed for their functionality. Metagenomics has now emerged as a very effective tool to mine for non‐redundant genes, creating panels of enzymes from the same families with low sequence homologies.[^14^, ^15^]

A sequence directed strategy increases the emphasis on *in silico* screening compared to other methods, using bioinformatics and enzyme functionality prediction to enhance the likelihood of the desired activity being displayed in the expressed enzymes. Alternative approaches incorporating metagenomic strategies directly assay enzymes expressed from their environmental hosts or as a recombinant protein in a more readily culturable host.[^16^, ^17^, ^18^] The enzyme functions are then experimentally determined, which requires high throughput assays which can be time consuming and costly. For example, in an interesting recent publication by Popovic *et al*. the activity of over 1 million fosmid and Lambda‐ZAP clones from 16 different environments was screened, generating 714 positive hits, 80 of which displayed validated esterase activity.^[19]^ Another commonly used strategy in metagenomic studies uses degenerate primers to amplify gene sequences of a target enzyme family from environmental samples. This can generate a larger number of enzymes per clones screened, however the enzymes often exhibit high sequence similarity to each other.^[20]^

The approach presented here follows our previously described metagenomics strategy, combining *in silico* protein function prediction with sequence specific PCR. This methodology increases the rate of positive hits that display the desired functionality, whilst at the same time delivering hits with comparatively low sequence similarity, which increases the chance of finding enzymes with different characteristics. Following this approach, 37 enzymes were expressed with low average sequence identity of 20–40%. Active enzymes were investigated for their substrate selectivity towards twenty substrates, including the Wieland Miescher ketone (WMK), a precursor to a wide selection of pharmaceutically relevant products. Selected enzymes from the metagenome were then used with the bicyclic WMK and co‐expressed with a cofactor recycling enzyme, glucose‐6‐phosphate dehydrogenase, to stereoselectively reduce the (*R*)‐WMK, in excellent yields.

## Results and Discussion

### Metagenome Mining and Isolation of SDR Genes

To identify potential SDR genes an *in silico* tongue metagenomic library was mined for enzymes using a method we have recently described.[^12^, ^13^] Such *in silico* libraries are a viable resource for the identification and retrieval of enzymes using both BLAST and Pfam based search methodologies.[^21^, ^22^] The Pfam ID for adh_short_C2 (PF13561.6) used to search the metagenome was generated when querying the Pfam database using amino acid sequences of SDRs from *Lactobacillus brevis* (Uniprot ID Q84EX5), *Lactobacillus kefiri* (Uniprot ID Q6WVP7), and *Weissella thailandensis* (Uniprot ID G0UH95). These were identified as sequences of interest as they exhibit preference for (*R*)‐alcohols.[^23^, ^24^] The sequences were also used in a BLAST search of the metagenomic library. In total 139 open reading frames (ORFs) were identified.

**Figure 1:**
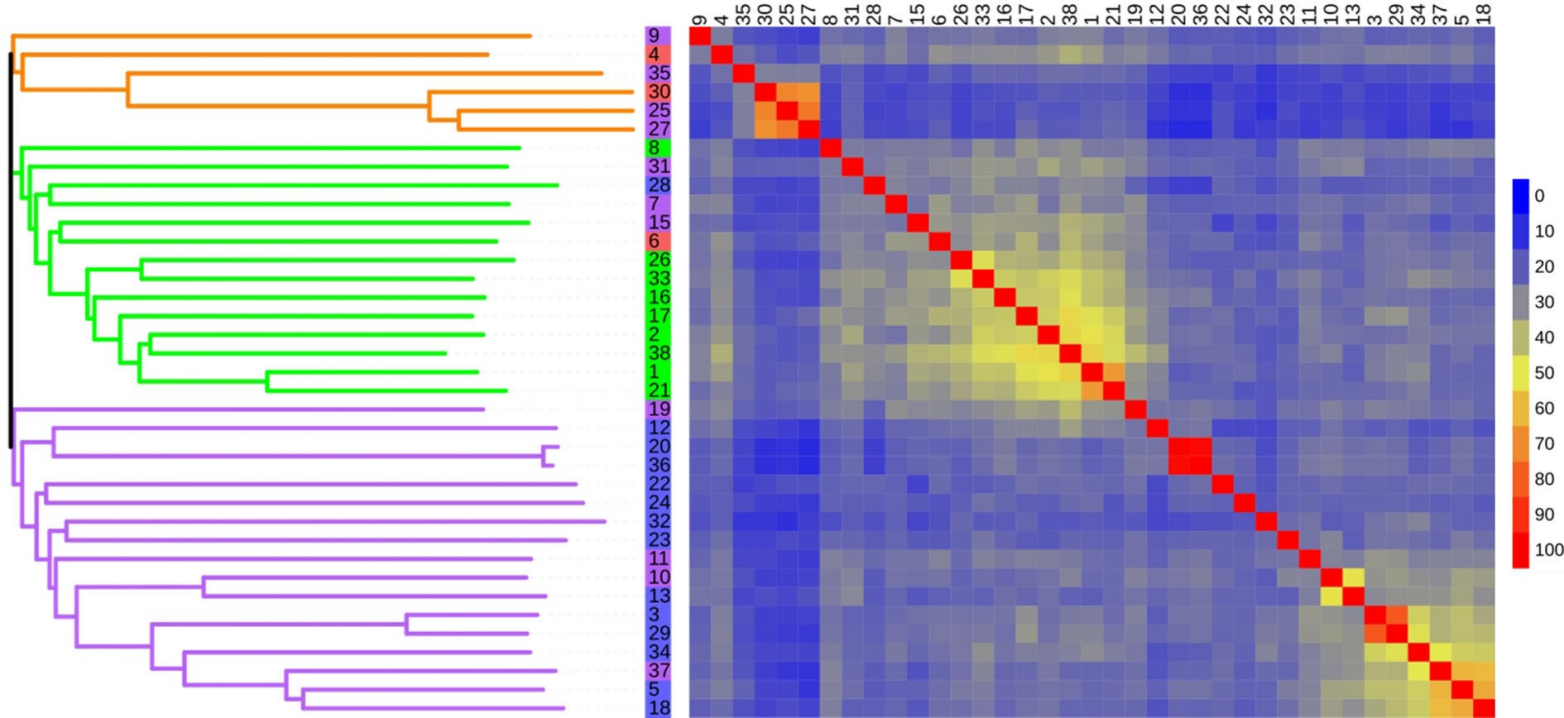
A heat map and phylogenetic tree showing the relationships and sequence identity of the metagenome derived SDRs. SDR numbers on the left are coloured to reflect their functional identification, SDR numbers in green were identified as 3‐oxo acyl ACP reductases, purple as NAD(P)H dependant oxidoreductases, blue as oxidoreductases and red were given unique functional assignments. Branches belonging to the same clade were coloured orange, green and purple.

From these ORFs, sequences were selected if they had an initiator methionine and stop codon and were at least 230–250 amino acids in length. They were clustered based on similarity and one from each cluster was chosen, giving 38 non redundant sequences, that were taken forward for cloning using Gibson assembly. All but one sequence, SDR‐14 were successfully amplified by PCR. Multiple sequence alignments of the 37 successfully retrieved SDRs showed sequence identity ranging from 11% to 98%, with most enzymes having identity to each other of between 20–40% (Figure 1). Some groups of enzymes had above average similarity suggesting that they may have similar properties such as: SDR‐10 and SDR‐13, 51%; SDR‐5, 18 and 37, ∼60%; SDR‐1 and SDR‐21, 67%; SDR‐25, 27 and 30, ∼70%; SDR‐3, SDR‐29, 81% and SDR‐20 and SDR‐36, 98%.

The protein and DNA sequences of the SDRs were searched against the NCBI database by BLAST. This generated predicted functions and taxonomic assignments. Enzyme functional assignments broadly fell into 3 groups, 3‐oxoacyl acyl carrier protein (ACP) reductases, NAD(P)H dependant oxidoreductases and oxidoreductases: SDR numbers are labelled in green, purple and blue respectively in Figure 1. The clade in green contains all the enzymes identified as 3‐oxoacyl ACP reductases but also those identified as oxidoreductases, SDR‐7, 15, 31 and a NAD(P)H dependant oxidoreductase SDR‐28. It is possible that these SDRs are also 3‐oxoacyl ACP reductases. This is possible as the three assignments are nested terms, that is, 3‐oxoacyl ACP reductases can be less specifically described as NAD(P)H dependant oxidoreductases and again as oxidoreductases. The clade in purple, contains only enzymes which were identified as NAD(P)H dependant oxidoreductases or oxidoreductases.

Three enzymes were given unique assignments, SDR‐4, 6 and 30 labelled in red. SDR‐4 was identified as an acetoin reductase, SDR‐6 as a glucose‐1‐dehydrogenase and SDR‐30 as an enoyl ACP reductase. Enzymes in the same clade as SDR‐30, in orange, were identified as oxidoreductases. It is possible that enzymes in this clade with high similarity, SDR‐25 and 27 could also be enoyl ACP reductases. Other enzymes in the clade but with lower similarity could similarly be enoyl ACP reductases, such as SDR‐35 or act on substrates with similar structure such as SDR‐4 the acetoin reductase (Figure 1). Taxonomic assignments came from multiple genera, all matched to known human oral commensals. *Streptococcus*, *Porphyromonas*, *Actinomyces*, *Haemophilus*, *Neiserria*, *Prevotella*, *Rothia* and *Veillonella* species were the source of multiple enzymes. Named species from these genera were the source of multiple enzymes, however of these multiple sequences, no more than one was mapped to the clade in green from any single species. *Rothia mucilaginosa* was the source of 4 enzymes SDR‐9, 10, 11 and 16: SDR‐10 and SDR‐11 map to the clade in purple while SDR‐16 maps to the clade in green. This pattern is repeated for *S. parasanguinis* with SDR‐5 and SDR‐24 mapping to the purple clade and SDR‐17 to the green clade (Figure 1). *Atopobium parvulum*, *Megasphaera micronuciformis, Oribacterium sinus* and *Mycobacteriodes abscessus* were the source of a single enzyme. The full taxonomic and functional assignments along with DNA and amino acid sequences are given in the supplementary information (SI, Tables S1‐3).

### Initial Screening of the SDR Enzymes

**Scheme 1:**
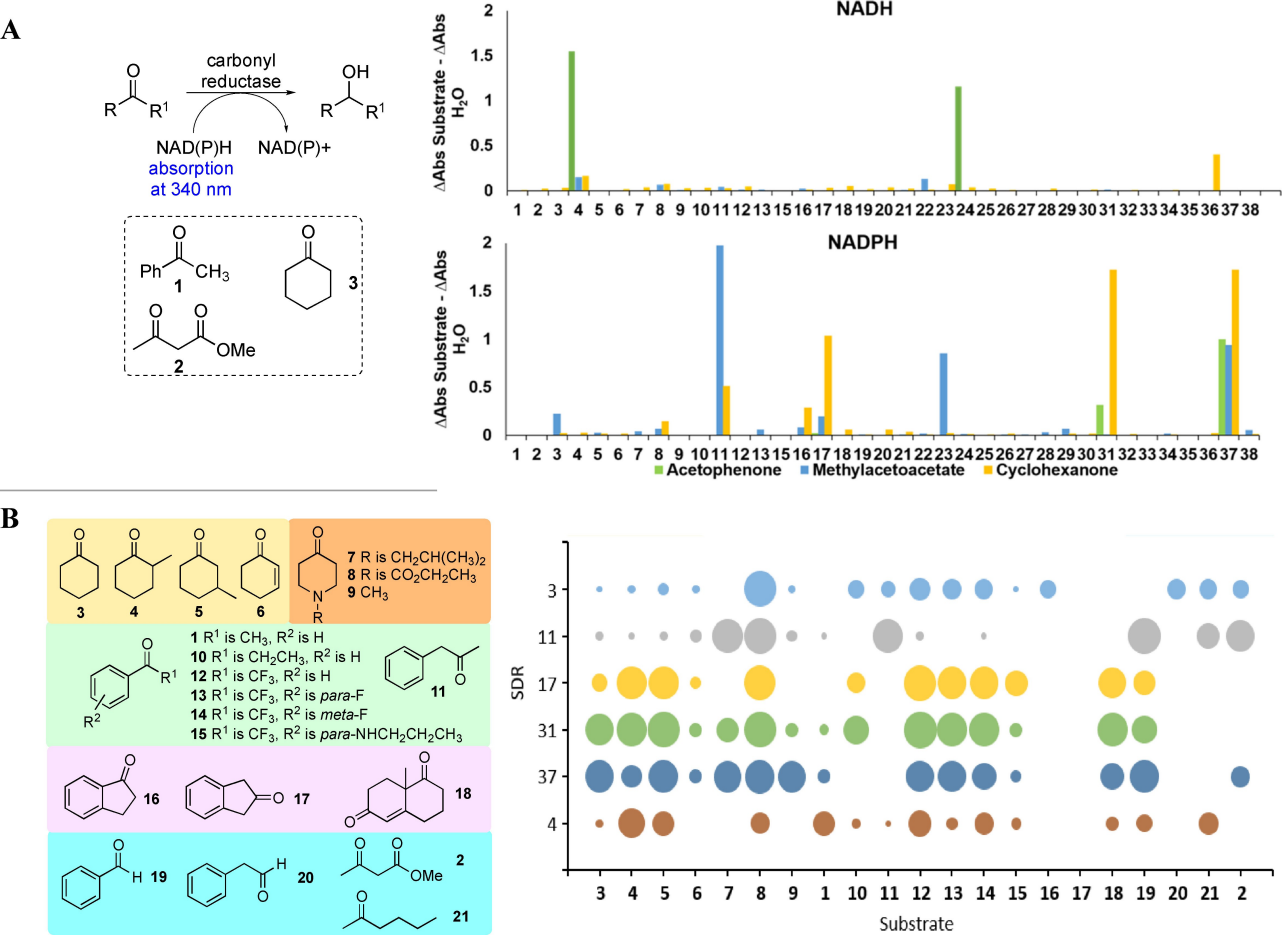
**A**. Spectrophotometric carbonyl reductase assay monitoring the consumption of NAD(P)H at 340 nm. The change in absorption (340 nm) after a 100 min incubation of substrates **1–3** with the 37 SDRs using either NADH (top) or NADPH (bottom) as co‐factor. *Reaction conditions*: substrate **1**, **2** or **3** (5 mM) NAD(P)H (1 mM) (200 μL), clarified cell lysate (0.4 mg/mL), KPi (100 mM, pH 7.2), DMSO (10%, v/v). The reactions were shaken for 100 min, at 25 °C, and quantified using the spectrophotometer at 340 nm. **B**. The change in absorption (340 nm) after a 100 min incubation of substrates **1–21** with SDRs‐3, 4, 11, 17, 31 and 37 using either NADPH or NADH as co‐factor. *Reaction conditions*: As for **A** and reactions were performed in triplicate, with an average standard deviation of ±0.05%.

The 37 successfully cloned SDRs were screened as clarified cell lysates against acetophenone **1**, methyl acetoacetate **2** and cyclohexanone **3**, using an NAD(P)H spectrophotometric assay (Scheme 1. A),^[25]^ to highlight initial activities. Notably, the SDRs had a clear preference for either NADPH or NADH. For example, acetophenone **1** was well accepted by SDR‐4 and SDR‐24 with NADH, while methyl acetoacetate **2** had low levels of acceptance by SDR‐4 and SDR‐22, and cyclohexanone **3** was accepted by SDR‐4 and SDR‐36 with the same co‐factor. With NADPH, **1** was accepted by SDR‐31 and SDR‐37, **2** predominantly by SDRs‐3, 11, 17, 23 and 37, and **3** by SDRs‐11, 16, 17 31 and SDR‐37. The most active enzymes displayed low sequence identity to each other in the range 20–40% (Figure 1).

Overall, from the 37 enzymes investigated, the success rate of finding an active enzyme towards the compounds tested was approximately 20%. Compared to related studies this was a higher hit rate and the retrieved enzymes were less homologous. For example, Itoh *et al*. used degenerate primers to identify ADHs from metagenomic material generated from 17 soil samples. They identified 240 putative ADH‐positive clones via colony PCR from 2800 colonies and only 10% functional ADH genes were collected that were very similar at the amino acid sequence level >90%.^[20]^

### Screening of Selected SDRs for Biocatalytic Applications

From the panel of enzymes, based upon the preliminary successful activity data and levels of expression, SDRs‐3, 11, 17, 31 and SDR‐37 were then screened against a larger substrate set, **1–21**, with NADPH and SDR‐4 with NADH, again with the spectrophotometric assay as described above. The results are summarised in Scheme 1B.

Reductases SDR‐17, SDR‐31 and SDR‐37 were the best performing enzymes, demonstrating high conversions on many of the substrates (up to 69%). The cyclohexanone ring systems (**3**‐**9**), were readily accepted by SDR‐17, SDR‐31, SDR‐37 and SDR‐4. Interestingly, the addition of a methyl group at C‐2 or C‐3 in **4** and **5** increased the acceptance by SDR‐4 and SDR‐17 compared to cyclohexanone **3**. For example, conversions increased from very low levels with **3** to 40% and 26% with SDR‐4 and **4** and **5** respectively. For SDR‐17, conversions increased from 11% with **3** to ∼55% with **4** and **5**. None of the enzymes screened showed much activity towards 2‐cyclohexen‐1‐one **6**, suggesting that they were poor acceptors of α,β‐unsaturated carbonyl functionalisation. Piperidone systems **7**, **8**, and **9**, were generally well accepted by the SDRs enzymes with up to 65% conversions. For the aromatic ketones, **1** and **10–15**, **1** was only readily accepted by SDR‐4 as indicated above, with **10** accepted by SDR‐31, and ketone **11** only readily accepted by SDR‐11. Substrates **12–14** were however, generally well accepted by SDRs‐3, 17, 31, 37 and SDR‐4 (>50% for SDRs‐17, 31 and SDR‐37), although **13** had the lowest activity. While these all possess aryl ketone‐CF_3_ groups, **13** has a *para*‐F moiety which may result in unfavourable interactions in the active site. In **15**, the electron donating *para*‐*N*‐propyl group will make the carbonyl carbon less electron deficient, reducing its reactivity, however unfavourable steric effects may have also lowered conversions with all the enzymes. For the bicyclic ketones, 1‐indanone **16** was only accepted at low levels by SDR‐3, while **17** was not accepted at all. Following these results, it was particularly interesting that the *rac*‐WMK **18** was well accepted by SDRs‐17, 31 and SDR‐37. Of the aldehydes tested, benzaldehyde **19** showed similar conversions to the aromatic ketones with SDR‐17, SDR‐31, and SDR‐37, but phenylacetaldehyde **20** was poorly accepted. For the linear substrate **21** it was accepted most by SDR‐11 as was **2**.

For these six SDRs showing the most interesting activities, the average sequence identity is 28%. Sequence alignment confirmed that the active site catalytic triad Ser‐Tyr‐Lys was conserved plus a fourth residue considered to be important. The TGxxxGxG motif important in cofactor binding was also present (SI Figure S1). Sequence analysis identified SDR‐4 as an acetoin reductase originating from *M. abseccus*, as well as having 99% sequence identity to a reported acetoin reductase from several *Streptococcus* species. Such reductases have been shown in the literature to reduce diacetyl and 2,3‐pentane‐dione as well as acetoin.^[26]^ However, they have not been reported to readily accept other linear ketones such as 2‐heptanone **21** or aromatic or cyclic ketones as noted here. SDR‐17 was identified as a 3‐oxoacyl‐ACP reductase involved in fatty acid synthesis and SDR‐31 as an oxidoreductase, although it was placed in the same clade as SDR‐17 in the phylogenetic tree constructed from all 37 sequences (Figure 1), suggesting it too may be a 3‐oxoacyl‐ACP reductase. 3‐Oxoacyl‐ACP reductases have been shown to exhibit a wide specificity with respect to the chain length of the β‐ketoacyl group.^[27]^ SDR‐17 and SDR‐31 also had wide substrate promiscuity, although no β‐ketoacyl‐ACP compounds were screened in this study. SDR‐37 although being identified as an NAD(P)H oxidoreductase and having similar substrate profiles to SDR‐17 and 31, has a low sequence identity of ∼24% with these enzymes and was placed in a different clade in the phylogenetic tree (Figure 1).

Considering the enzymes, in general SDR‐3 accepted aromatic ketones, but surprisingly the cyclic ketone **8** at high levels. SDR‐3 was also the only enzyme to accept substrates **16** and **20**. SDR‐11, was also active towards fewer compounds but tolerated some of the substrates that were poorly accepted by the other tested enzymes, such as **2** and **11**. SDR‐17, SDR‐31 and SDR‐37 had the best substrate tolerances towards the panel of compounds, while SDR‐4 accepted approximately half of the substrates.

### Development of the WMK Substrate with Selected SDRs

**Scheme 2:**
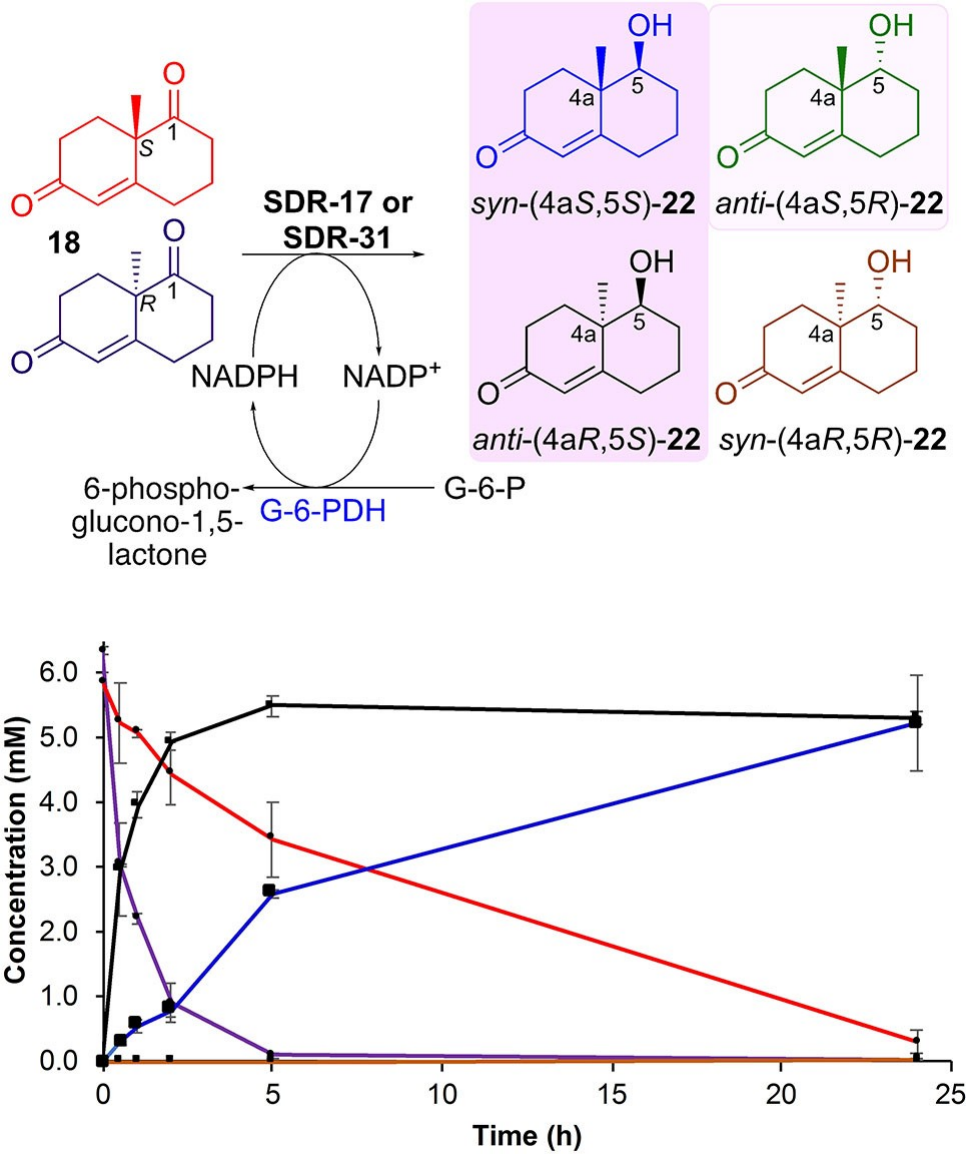
Consumption of *rac*‐**18** with SDR‐17 co‐expressed with G‐6‐PDH and production of **22**. *Reaction conditions*: (500 μL volume): *rac*‐**18** (12 mM), clarified cell lysate (0.4 mg/mL), NADP^+^ (3 mM), glucose‐6‐phosphate (G‐6‐P) (100 mM), KPi (100 mM, pH 7.2), DMSO (10%, v/v). The reaction was shaken for 24 h at 37 °C. Reactions were performed in duplicate. Red (*S*)‐**18**, purple (*R*)‐**18**, products blue *syn*‐(4a*S*,5*S*)‐**22**, black *anti*‐(4a*R*,5*S*)‐**22**, green *anti‐*(4a*S*,5*R*)‐**22** and brown *syn*‐(4a*R*,5*R*)‐**22** were formed.

The WMK **18** is a precursor to many natural products and analogues which have medical applications as anti‐inflammatory, anti‐fungal and anti‐cancer treatments.^[28]^ The regioselective reduction at the C‐1 carbonyl in **18** has been reported using sodium or zinc borohydride.[^29^, ^30^] For example, the reduction of *S*‐**18** using sodium borohydride was incorporated into the total synthesis of baccatin III to give (4a*S*,5*S*)‐**22** with a stereoselective reduction at the least hindered face of the bicycle, resulting in a *syn*‐relationship between the methyl group at C‐4a and C‐5 OH.^[31]^ There have been limited investigations into the use of biocatalysts to perform the reduction of **18** with varying yields and stereoselectivities. Notably, whole cell experiments with the yeasts *Torulaspora delbrueckii* which preferentially reduced (*S*)‐**18** (70% *e.e*.) to give *syn*‐(4a*S*,5*S*)‐**22** in 26% yield, while *Candida melibiosica* reduced (*R*)‐**18** to give *anti*‐(4a*R*,5*S*)‐**22** in 31% yield.^[32]^ Baker's yeast has also been utilised to reduce **18** and gave *syn*‐(4a*S*,5*S*)‐**22** as the major product in 32% yield together with 4% of *anti*‐(4a*R*,5*S*)‐**22**,^[33]^ and using *Coryneum betulinum* or *Didymosphaeria igniaria* cultures also preferentially converted (*S*)‐**18** into *syn*‐(4a*S*,5*S*)‐**22**: reduction of (*R*)‐**18** was slow and required 4–6 days to give *anti*‐(4a*R*,5*S*)‐**22** in only moderate diastereoselectivities (46‐64% *d.e*.).^[34]^ Other approaches have involved enzymatic resolutions of acetylated‐**22** with commercial lipases.^[35]^ It was hoped that following the promising levels of reactivity of SDR‐17 and SDR‐31 towards **18**, the metagenomic‐derived recombinant enzymes could be used to selectively produce isomers of **22** (Scheme 2), particularly those that are more difficult to access.

Initially, studies were performed using SDR‐17 and SDR‐31 with either (*S*)‐**18** or (*R*)‐**18** at different pHs using the spectrophotometric assay. Both SDR‐17 and SDR‐31 were more active at pH 7–8 and this was used in later reactions (SI Figure S2). In addition, SDR‐17 displayed lower activity towards (*S*)‐**18**, while SDR‐31 readily accepted both (*S*)‐ and (*R*)‐**18**, with a preference for the (*S*)‐isomer.

When performing non‐whole cell biocatalytic reactions that require co‐factors either these must be supplied directly, which is possible on small scales, or co‐factor recycling systems are required. These can be produced separately, however co‐expression can enhance the ease of use. Glucose‐6‐phosphate dehydrogenase (G‐6‐PDH) was selected as a co‐factor recycling system due to its high activity towards NADP^+^. Co‐expression was successful with both SDR‐17 and SDR‐31 and using *rac*‐**18** quantitative conversions were achieved with NADP^+^ at 37 °C after 24 h. Notably, SDR‐17 was very stereoselective and only produced the *S*‐enantiomer at C‐5, *syn*‐(4a*S*,5*S*)‐**22** and *anti*‐(4a*R*,5*S*)‐**22**, and the time course is shown in Scheme 2. This revealed the more rapid consumption of (*R*)‐**18**. Moreover, under the same conditions SDR‐31 fully reduced *rac*‐**18** after 24 h, with the (*S*)‐isomer converted into *syn*‐(4a*S*,5*S*)‐**22** only and the (*R*)‐isomer generating mostly *anti*‐(4a*R*,5*S*)‐**22** with a small amount of *syn*‐(4a*R*,5*R*)‐**22** in a ratio of ∼6:5:1, respectively. Reaction stereoselectivities were determined by the reduction of (*R*)‐**18** and (*S*)‐**18** using sodium borohydride to give (predominantly) the *syn*‐products as previously described.^[30]^ A combination of chiral HPLC and NMR spectroscopy confirmed the absolute stereochemistry in the products.

To explore the utility of the enzyme system further, SDR‐17 co‐expressed with G‐6‐PDH (as a crude cell lysate) was used to perform a preparative enzyme scale reaction using (*R*)‐**18** (20 mM substrate, 50 mL reaction) which generated *anti*‐(4a*R*,5*S*)‐**22** in 89% isolated yield after 24 h and >99% *e.e*. (at C‐5). Notably, the product was readily isolated in high purity without the use of chromatographic methods, by extraction with ethyl acetate. The reaction was highly stereoselective giving the *anti‐*product which is less readily produced using traditional borohydride reducing reagents. Since the SDR has been successfully co‐expressed with a co‐factor recycling system, further scale‐up has the potential for delivery of an economical and efficient bioreduction process for multiple carbonyl containing substrates yielding the subsequent alcohol.

## Conclusion

The metagenomic approach utilized here is an effective tool for searching for new enzymes. Using bioinformatics to predict reductase functionality from a searchable metagenomic library, yielded 37 enzymes of high sequence diversity (20–40% sequence identity). Compared with other metagenomic methods this is a higher hit rate for active enzymes, whilst also generating sequences with much lower overall similarity.[^19^, ^20^]

From the 37 retrieved enzymes, expression and screening highlighted several (∼20%) with interesting activity profiles. The panel of 6 enzymes taken through for further investigation showed good coverage against the 21 substrates tested, including bulkier and more lipophilic substrates that have been noted as challenging for biocatalytic reductions.^[7]^ SDR‐17 and SDR‐31 showed interesting activities toward monocyclic ketones and for the bicyclic ketone **18**, a useful synthetic precursor, the pH preference of the enzymes was also established and the timecourse for the reaction monitored. SDR‐17 was then used for the reduction of (*R*)‐**18** in high yields and excellent stereoselectivity at a 50 mL scale. Moreover, the product *anti*‐(4a*R*,5*S*)‐**22** was readily isolated by extraction from the reaction media.

In this work one enzyme family, the SDRs were investigated, however there are other potential carbonyl reducing enzyme families which could be investigated, such as aldo keto reductases, alcohol dehydrogenases, aldehyde dehydrogenases and iron and zinc containing alcohol dehydrogenases. Expanding the scope of enzyme families targeted and applying the technique to more diverse environmental metagenomes, has the potential to generate many more enzymes of interest.

## Experimental Section

### General experimental

All reagents were obtained from commercial sources (Sigma Aldrich, Fisher, Alfa Aesar) and used as received unless otherwise stated. Silica column chromatography was performed using Geduran® Si 60 Silica (43–60 μM). Thin layer chromatography was performed using plates with a silica gel matrix on an aluminium support. Infrared spectroscopy was carried out using a Spectrum 100 FTIR spectrometer (Perkin‐Elmer). ^1^H and ^13^C NMR spectra were obtained using an Avance 600 (Bruker) spectrometer. Chemical shifts specified are relative to trimethylsilane (set at 0 ppm) and referenced to the residual, protonated NMR solvent. Coupling constants in ^1^H NMR spectra (*J*) are given in Hertz (Hz) and described as singlet (s), doublet (d), multiplet (m). Mass spectroscopy was carried out using a VG70‐SE mass spectrometer Trace 1310 Gas Chromatograph (Thermoscientific) connected to a ISQ single quadrapole MS (Thermoscientific). Melting points were determined using IA9000 Series melting point apparatus (Electrothermal). Analytical HPLC analysis was carried out using a Series 1100 (Hewlett Packard) or 1260 (Aligent Technologies) instruments with a HiChrom ACE C18 column (250 mm×4.6 mm) or Chiralcel OJ column (250×4.6 mm).

Sonication of cell lysates was performed using a probe Soniprep 150 (MSE) sonicator. The following centrifuges were used: Allegras x‐15R centrifuge (Beckman Coulter), Centrifuge 5415R (Eppendorf), Centrifuge 5810R (Eppendorf), Centrifuge 5430R (Eppendorf). A ShakerX ClimoShaker ISFI‐X (Kuhner), Innova 44 (New Brunswick Scientific) or Mixing Block MB‐102 (BIOER) shaker was used. A GENios plate reader (Tecan) was used. Chemicals, media and apparatus were autoclaved (Priorclave) at 121 °C for 30 min where required.

### Cloning of SDR Sequences

Primers matching the 38 target sequences were designed and, appended to these primers, were sequences identical to the DNA sequence of the expression vector pET29a immediately upstream and downstream of its multiple cloning site. The pET29a sequences appended to the forward primers were chosen to facilitate the insertion of the amplified genes with the initiator methionine at the translation start site in the expression vector. The reverse primers were designed to remove the endogenous stop codon and allow read through of the hexa‐histidine tag encoded by the expression vector. Gibson assembly (HiFi assembly NEB) was used to include the PCR products of the SDR genes directly into pET29a plasmids. Amplifications of genes from the metagenome and cloning into the vector were successful for 37 out of 38 target sequences. Primers and adapters for Gibson cloning are given in in the SI Table S4. The recombinant vectors were then transformed into a chemically competent cloning strain *E. coli* Top10 (Invitrogen) and a chemically competent expression strain *E. coli* BL21*(DE3)pLysS (Invitrogen) and stored as glycerol stocks.

### SDR Growth, Purification and Desalting

Glycerol stocks of the expression strains were used to inoculate Terrific Broth (TB) medium (Merck, 500 μL/well in a 96 deep‐square well plate) containing kanamycin (50 μg/mL) and chloramphenicol (30 μg/mL) and incubated for 16 h, 37 °C, 400 rpm (sealed with a microporous breathable membrane). Then 100 μL of this starter culture was added to TB media (10 mL) containing kanamycin (50 μg/mL) and chloramphenicol (30 μg/mL). This was incubated for 6–8 h, until an OD_600_ of 0.6–0.8 was achieved. IPTG (1 mM final concentration) was added and the mixture incubated for 16 h at 25 °C at 400 rpm. Cells were harvested by centrifugation (12000 rpm, 4 °C, 5 min) and resuspended in lysis buffer (1 mL) containing BugBuster protein extraction solution (Novagen), DNAse I (20 μL/mL) and lysozyme (1 μg/mL). The cell lysate was aliquotted into 20 μL volumes and flash frozen at −80 °C for storage. Where applicable, NTA−Ni spin columns (QAIGEN) were used to purify the protein, following the manufacturer's instructions. The protein was buffer exchanged into 0.1 M sodium phosphate buffer pH 7.2 using Zebra Spin Desalting plates (Thermoscientific) following the manufacturer's instructions.

### SDS‐PAGE Procedures

The protein composition of induced cells was analysed by sodium dodecyl sulfate‐12% polyacrylamide gel electrophoresis (SDS‐PAGE) using Mini‐Protean TGX Gels (Bio‐Rad). Protein samples were prepared by heating for 5 min at 95 °C in the presence of sample buffer (1:1 dilution of 2x Laemmli Sample Buffer (Bio‐Rad) and 100 mM dithiothreitol:protein). A broad range protein marker (10–25 kDa, New England Biolabs) was used to estimate the molecular mass of the proteins. Imperial Protein Stain (Thermoscientific) was used to stain the protein (SI Figure S3).

### Spectrophotometric SDR Assay

Activity data on the SDR reduction of ketones was determined at room temperature by following the oxidation of NAD(P)H using a GENios microplate reader (Tecan) at 340 nm over 100 cycles of 57 seconds with a shake duration of 1 s and a shake settle time between cycles of 1 s. Each reaction mixture (200 μL) contained 10 mM sodium phosphate buffer (pH 7.2), 5 mM substrate, DMSO (10%, v/v) and the enzyme lysate (20 μL). The reaction was initiated by the addition of the substrate. Protein concentrations were determined using a Nanodrop 2000c spectrophotometer (Thermoscientific).

### Co‐Expression of SDRs and G‐6‐PDH

Competent *E. coli* BL21 DE3 cells were transformed using standard protocols with the SDR pET29a plasmid (3 μL) and G‐6‐PDH from *Saccharomyces cerevisiae* SF838 (Uniprot P11412) in a pACYCDuet‐1 plasmid (3 μL). The SDR and G‐6‐PDH were then expressed as previously described above.

### Stereoselectivities and the WMK Reactions

Reaction conversion yields and enantiomeric excesses at the reduced centre were determined by chiral HPLC using a Chiracel OJ column. For 200 μL scale reactions, the assay procedure above was followed, using flash‐frozen clarified cell lysate. For 500 μL scale reactions using co‐ expressed SDR‐17 and SDR‐31 with G‐6‐PDH, enzymes were expressed as described above, and freeze‐dried. Each reaction mixture (500 μL) contained 10 mM sodium phosphate buffer (pH 7.2), 10 mM (*R*)‐ or (*S*)‐**18**), DMSO (10%, v/v) and the 0.4 mg/mL SDR enzyme. The reaction was initiated by the addition of the substrate. The reaction was shaken (250 rpm, 24 h, 25 °C). The reaction was stopped by the addition of TFA (0.5% v/v) and denatured protein was removed by centrifugation. Diethyl ether (1 mL) was then added to the supernatant and the mixture vortexed for 30 s. The organic layer was separated, dried (Na_2_SO_4_) and evaporated. EtOH (200 μL) was then added and the mixture analysed by analytical HPLC (Chiralcel OJ column). Concentrations of substrates and products were determined using calibration curves against standards, SI Figure S4, (solvents: 6% 2‐propanol/hexane at 0.5 mL/min flow‐rate, detection at 230 nm and a run time of 120 min). Retention times: (*R*)‐**18** 68.2 min; (*S*)‐**18** 78.3 min; (4a*S*,5*S*)‐**22** 85.9 min; (4a*R*,5*R*)‐**22** 61.2 min; (4a*R*,5*S*)‐**22** 54.0 min.

#### (4a*R*,5*R*)‐5‐Hydroxy‐4 a‐methyl‐4,4 a,5,6,7,8‐hexahydronaphthalen‐2(3*H*)‐one, (4a*R*,5*R*)‐22^[36]^

To a stirred solution of (*R*)‐**18** (0.020 g, 0.12 mmol) in EtOH (500 μL) at 0 °C, NaBH_4_ (5.5 mg, 0.11 mmol) was added and the reaction stirred for 15 min. Acetic acid (50 μL) was added and the reaction stirred for 15 min at 0 °C. The solvents were evaporated, and the remaining mixture was extracted with EtOAc (3×10 mL) and washed with sat. NaCl solution (2×10 mL). The organic layer was dried (MgSO_4_) and concentrated *in vacuo*. The crude product was purified using a silica plug and washed with EtOAc (10 mL) to give (4a*R*,5*R*)‐**22** as an oil (0.019 g, 96%), 98% *d.e*. by chiral HPLC analysis (ratio 99:1, 4a*R*,5*R*:4a*R*,5*S*). R_f_ 0.30 (30% EtOAc in 40–60 petroleum ether); *ν*_max_ (neat) 3407, 2932, 1719, 1614 cm^−1^; ^1^H NMR (600 MHz; CDCl_3_) δ 5.80 (1H, d, *J*=1.9 Hz, 1‐H), 3.44 (1H, br d, *J*=11.7 Hz, 5‐H), 2.31–2.50 (3H, m, 3‐H_2_, 8‐*H*H), 2.16–2.26 (2H, m, 4‐*H*H, 8‐H*H*), 1.81–1.94 (3H, m, 4‐H*H*, 6‐*H*H, 7‐*H*H), 1.66–1.76 (1H, m, 6‐H*H*), 1.37–1.48 (1H, m, 7‐H*H*),1.21 (3H, s, CH_3_); ^13^C NMR (150 MHz; CDCl_3_) δ 199.6, 168.3, 125.7, 78.5, 41.7, 34.4, 33.8, 32.1, 30.4, 23.3, 15.4; *m/z* (EI) 180 ([M]^+^). Analytical chiral HPLC retention time 61.2 min (Chiralcel OJ, propanol/hexane at 0.5 mL/min; SI Figure S4).

#### (4a*R*,5*S*)‐5‐Hydroxy‐4 a‐methyl‐4,4 a,5,6,7,8‐hexahydronaphthalen‐2(3*H*)‐one, (4a*R*,5*S*)‐22

Freeze‐dried cells of SDR‐17 (25 mg) were suspended in buffer (NaPi, 100 mM, 1 mL) and sonicated (10×15 s pulses) at 0 °C before being centrifuged (10 min, 3000 g, 4 °C). The reaction was performed using a total volume of 50 mL, containing buffer (NaPi, 100 mM, pH 7.2), (*R*)‐**18** (20 mM), clarified cell lysate (0.4 mg/mL), NADP^+^ (3 mM), DMSO (10%, v/v) and G‐6‐P (100 mM), and shaken for 24 h, 37 °C, at 500 rpm. The reaction was stopped with the addition of TFA (0.5% v/v), and the product was extracted with EtOAc (5×15 mL) and washed with sat. NaCl solution (5×15 mL) to afford (4a*R*,5*S*)‐**22** as a yellow solid (0.160 g, 89%, >99% *e.e*. by HPLC analysis). M.p. 75–79 °C [lit. 88–90 °C^[37]^]; R_f_ 0.61 (33% EtOAc in 40–60 petroleum ether); [α]^25^_D_ −112 (c 1.3, toluene) [lit. [α]^25^_D_ −111 (c 1.3, benzene)];^[38]^ *ν*_max_ (neat) 3427, 2963, 1638, 1600 cm^−1^; ^1^H NMR (600 MHz; CDCl_3_) δ 5.87 (1H, d, *J*=1.9 Hz, 1‐H), 3.65 (1H, t, *J*=2.7 Hz, 5‐H), 2.56–2.62 (1H, m, 4‐*H*H), 2.44–2.52 (2H, m, 3‐H_2_), 2.39–2.44 (1H, m, 8‐*H*H), 2.24–2.32 (1H, m, 8‐H*H*), 2.01–2.09 (1H, m, 6‐*H*H), 1.82–1.93 (1H, m, 7‐*H*H), 1.76–1.82 (1H, m, 6‐H*H*), 1.67–1.75 (1H, m, 7‐*H*H), 1.47–1.53 (1H, m, 4‐H*H*), 1.24 (3H, s, CH_3_); ^13^C NMR (150 MHz; CDCl_3_) δ 199.6, 168.0, 127.2, 75.4, 41.0, 34.1, 31.8, 30.9, 28.8, 21.9, 19.9; *m/z* (ES+) 181 ([M+H]^+^). Analytical HPLC retention time 54.0 min (Chiralcel OJ, propanol/hexane at 0.5 mL/min; SI Figure S6).

#### (4a*S*,5*S*)‐5‐Hydroxy‐4 a‐methyl‐4,4 a,5,6,7,8‐hexahydronaphthalen‐2(3*H*)‐one, (4a*S*,5*S*)‐22^[39]^

To a stirred solution of (*S*)‐**18** (0.030 g, 0.17 mmol) in EtOH (500 μL) at 0 °C, NaBH_4_ (2.0 mg, 0.057 mmol) was added and the reaction stirred for 15 min. The solvents were evaporated *in vacuo*, and the mixture extracted with EtOAc (3×10 mL) and washed with sat. NaCl solution (2×10 mL). The organic layer was dried (MgSO_4_) and concentrated *in vacuo*. The product was purified via flash silica column chromatography (50% EtOAc in 40–60 petroleum ether) to give (4a*S*,5*S*)‐**22** as a colourless oil (0.019 g, 63%), 96% *d.e*. by chiral HPLC analysis (ratio 98:2 (4a*S*,5*S*):(4a*S*,5*R*)). R_f_ 0.32 (50% EtOAc in 40–60 petroleum ether); [α]^25^_D_ +115 (c 0.18, CHCl_3_) [lit. [α]^25^_D_ +123 (c 0.34, CHCl_3_)];^[39]^ *ν*_max_ (neat) 3320, 2971, 1655 cm^−1^; ^1^H NMR (600 MHz; CDCl_3_) δ 5.79 (1H, d, *J*=1.8 Hz, 1‐H), 3.44 (1H, dd, *J*=11.7, 4.2 Hz, 5‐H), 2.30–2.49 (3H, m, 3‐H_2_, 8‐*H*H), 2.15–2.26 (2H, m, 4‐*H*H, 8‐H*H*), 1.81–1.94 (3H, m, 4‐H*H*, 6‐H*H*, 7‐H*H*), 1.63–1.76 (2H, m, 6‐H*H*, OH), 1.37–1.48 (1H, m, 7‐*H*H), 1.20 (3H, s, CH_3_); ^13^C NMR (150 MHz; CDCl_3_) δ 199.7, 168.4, 125.7, 78.5, 41.7, 34.4, 33.8, 32.1, 30.4, 23.3, 15.4; *m/z* (EI) 180 ([M]^+^). Analytical chiral HPLC retention time 85.9 min (Chiralcel OJ, propanol/hexane at 0.5 mL/min; SI Figure S5).

## Supporting information

As a service to our authors and readers, this journal provides supporting information supplied by the authors. Such materials are peer reviewed and may be re‐organized for online delivery, but are not copy‐edited or typeset. Technical support issues arising from supporting information (other than missing files) should be addressed to the authors.

Supplementary
