# Supplementary material for: Discovery of New Carbonyl Reductases Using Functional Metagenomics and Applications in Biocatalysis
Source: Adv Synth Catal. 2021 May 4;363(12):3044–52. doi: 10.1002/adsc.202100199 (PMC8360200; doi:10.1002/adsc.202100199)

Supporting Information

Discovery of New Carbonyl Reductases Using Functional Metagenomics and Applications in Biocatalysis

Sophie A. Newgas, Jack W. E. Jeffries, Thomas S. Moody, John M. Ward,* and Helen C. Hailes*© 2021 The Authors. Advanced Synthesis & Catalysis published by Wiley-VCH GmbH. This is an open access article under the terms of the Creative Commons Attribution License, which permits use, distribution and reproduction in any medium, provided the original work is properly cited.

Supplementary Information

Discovery of new carbonyl reductases using functional metagenomics and applications in biocatalysis

Sophie A. Newgas,[a] Jack W. E. Jeffries,[b] Thomas S. Moody,[c,d] John M. Ward[b]* and Helen C. Hailes[a]*

[a] Department of Chemistry, University College London, 20 Gordon Street, London, WC1H 0AJ, U.K. E-mail: h.c.hailes@ucl.ac.uk
[b] Department of Biochemical Engineering, Bernard Katz Building, University College London, London, WC1E 6BT, U.K. E-mail: j.ward@ucl.ac.uk
[c] Almac Sciences, Department of Biocatalysis and Isotope Chemistry, Almac House, 20 Seagoe Industrial Estate, Craigavon, BT63 5QD, Northern Ireland, U.K.
[d] Arran Chemical Company, Unit 1 Monksland Industrial Estate, Athlone, Co. Roscommon, Ireland.

Table of Contents

1. **Tables**

Table S1. The taxonomic and enzymatic assignments of the SDRs retrieved from the metagenome.

Table S2. DNA sequences of the 37 retrieved SDRs generated by sanger sequencing after Gibson assembly into pET29a.

Table S3. Amino acid sequences of the 37 retrieved SDRs.

Table S4. Table of primers used to retrieve SDRs from the oral metagenome.

2. **Figures**

Figure S1. A. Sequence alignments for SDRs 3,4,11,17,31,37. **B**. SDR percentage identity matrix for 3,4,11,17,31,37.

Figure S2. Graph displaying activity with (*S*)-**18** and (*R*)-**18** with SDR-17 and SDR-31 at a range of pHs.

Figure S3. SDS PAGE showing induced recombinant protein expression in *E.coli*.

Figure S4. HPLC calibration curves.

Figure S5. HPLC trace of the products from the reduction of *rac*-WMK-**18** using $NaBH_4$.

Figure S6. Characterisation data for (4a*R*,5*S*)-**22** from the scaled-up reaction using SDR-17.

Figure S7. ^{1}H and ^{13}C NMR spectra of (4a*R*,5*R*)-**22.**

Figure S8. ^{1}H and ^{13}C NMR spectra of (4a*S*,5*S*)-**22.**

Table S1. The taxonomic and enzymatic assignments of the SDRs retrieved from the metagenome. Assignments were generated by inputting the amino acid sequences of the SDRs into NCBI BLASTP. pQR numbers were assigned to recombinant plasmids for record keeping and identification within the UCL enzyme library.

1	pQR500	3-oxoacyl ACP reductases	*Porphyromonas somerae*
2	pQR501	3-oxoacyl ACP reductase	*Neiserria.sp*
3	pQR502	NAD(P)-dependent oxidoreductase	*Veillonella sp.*
4	pQR503	acetoin reductase	*Mycobacteroides abscessus*
5	pQR504	NAD(P)-dependent oxidoreductase	*Streptococcus parasanguinis*
6	pQR505	glucose-1-dehydrogenase	*Megasphaera micronuciformis*
7	pQR506	oxidoreductase	*Veillonella sp*
8	pQR507	3-oxoacyl ACP reductase	*Streptococcus infantis*
9	pQR508	oxidoreductase	*Rothia mucilaginosa*
10	pQR509	oxidoreductase	*Rothia mucilaginosa*
11	pQR510	oxidoreductase	*Rothia mucilaginosa*
12	pQR511	NAD(P)-dependent oxidoreductase	*Actinobacteria*
13	pQR512	NAD(P)-dependent oxidoreductase	*Actinomyces odontolyticus*
15	pQR513	oxidoreductase	*Atopobium parvulum*
16	pQR514	3-oxoacyl ACP reductase	*Rothia mucilaginosa*
17	pQR515	3-oxoacyl ACP reductase	*Streptococcus parasanguinis*
18	pQR516	NAD(P)-dependent oxidoreductase	*Neisseria mucosa*
19	pQR517	oxidoreductase	*Streptococcus sp*
20	pQR518	NAD(P)-dependent oxidoreductase	*Prevotella melaninogenica*
21	pQR519	3-oxoacyl ACP reductase	*Prevotella sp.*
22	pQR520	NAD(P)-dependent oxidoreductase	*Prevotella sp*
23	pQR521	NAD(P)-dependent oxidoreductase	*Prevotella sp*
24	pQR522	NAD(P)-dependent oxidoreductase	*Streptococcus parasanguinis*
25	pQR523	NAD(P)-dependent oxidoreductase	*Prevotella pallens*
26	pQR524	3-oxoacyl ACP reductase	*Neisseria sp*
27	pQR525	NAD(P)-dependent oxidoreductase	*Prevotella salivae*
28	pQR526	NAD(P)-dependent oxidoreductase	*Oribacterium sinus*
29	pQR527	NAD(P)-dependent oxidoreductase	*Veillonella atypica*
30	pQR528	enoyl-(acyl-carrier-protein)	*Porphyromonas sp*
31	pQR529	oxidoreductase	*Actinomyces graevenitzii*
32	pQR530	NAD(P)-dependent oxidoreductase	*Prevotella sp*
33	pQR531	3-oxoacyl ACP reductase	*Prevotella sp*
34	pQR532	NAD(P)-dependent oxidoreductase	*Prevotella histicola*
35	pQR533	oxidoreductase	*Haemophilus parainfluenzae*
36	pQR534	NAD(P)-dependent oxidoreductase	*Prevotella melaninogenica*
37	pQR535	oxidoreductase	*Haemophilus influenzae*
38	pQR536	3-oxoacyl ACP reductase	*Veillonella sp*

Table S2. DNA sequences of the 37 retrieved SDRs generated by sanger sequencing after Gibson assembly into pET29a. Sequences in italics originate from pET29a MCS, coding for 20 amino acids plus the hexa histidine tag.

SDR #	DNA sequence
1	ATGAATCTACTTGCTAACAAGGTAGCCATCATCACCGGCGCAGGCCGTGGTATCGG<br>CCGCGCTATTGCACTCAAGTATGCACAGGAAGGTGCTTCCGTAGTGATCACTGACCT<br>CAAGATCGACGAGACTGTAGAAGCCTTCGTCAAGGAGCTCGAAGGGCTCGGTGTCA<br>AGGCTAAGGCTTATGCCTCGAACGCAGCTAACTTCGAAGATGCTCACAAGCTCGTC<br>GAAGCAGTCGTCGCAGACTTCGGCCGTATCGACGTCCTCGTCAACAACGCTGGTAT<br>CACCCGTGACGGGCTGATGATGCGTATGACCGAAGAGCAGTGGGATCTCGTCATCA<br>ACGTCAACCTCAAGAGTGCCTTCAACCTCATCCACGCTGTCACCCCCGTTATGGTCA<br>AGCAGCGTAGCGGTAGCATCATCAACATGGCCAGCGTCGTCGGTGTCTCTGGCAAC<br>GCAGGTCAGGCGAACTACTCCGCTTCTAAGGCTGGTATGATCGGTCTGGCAAAGAG<br>CATCGCAAAGGAGCTCGGCGCTCGTGGTATCCGTGCCAACGCTATCGCTCCTGGCTT<br>CATCATCACCGATATGACGGGTGCACTCTCTGAAGAAGTTCGCAAGCAGTGGGAAG<br>TACAGATCCCCCTCCGTCGTGGCGGTACGCCTGAGGACGTAGCTAACGTAGCTACC<br>TTCCTCGCAAGCGACCTCTCCAGCTACGTCTCCGGTCAGACGATCCACGTCTGCGGT<br>GGGATGAATATG*CCATGGCTGATATCGGATCCGAATTCGAGCTCCGTCGACAAGCTTGC*<br>*GGCCGCACTCGAGCACCACCACCACCACCA*
2	ATGAGTACACAAGATTTGAGCGGCAAAATTGCTTTGGTAACCGGCGCATCGCGCGG<br>TATTGGTGCGGCGATTGCCGATACTTTGGCTGTGGCCGGTGCAAAAGTTATCGGCAC<br>GGCCACCAGCGAAAGCGGCGCGGCTGCAATTAGCGAGCGTCTGGCGCAATGGGGC<br>GGCGAAGGCCGTGCATTGAATTCCGCCGAACCCGAAACCATCGAAAACCTGATCGC<br>CGACATCGAAAAAGAGTTCGGCAAGCTGGATATTCTGGTCAACAACGCCGGTATCA<br>CCCGCGACAACCTCTTGATGCGTATGAAAGAAGAAGAGTGGGACGACATCATGCAG<br>GTCAACCTCAAATCCGTGTTCCGCGCCTCCAAAGCCGTCTTGCGCGGCATGATGAA<br>GCAACGCGCCGGCCGCATCATCAACATCACATCCGTTGTCGGTGTGATGGGCAATG<br>CCGGTCAAACCAACTATGCCGCGGCAAAAGCGGGCTTAATCGGTTTCTCCAAATCC<br>ATGGCGCGTGAAGTCGGCAGCCGCGGCATTACCGTCAACTGCGTCGCCCCAGGCTT<br>TATCGACACCGATATGACCCGCGCCCTGCCGGAAGAAACCCGCAAAACCTTTGAAG<br>CGCAAACTTCTTTGGGCAAATTCGGTGAAGCGCAAGATATTGCTGATGCAGTCTTGT<br>TCTTGGCTTCCGATCNGCAAAATACATTACCGGTCAGACACTTCATGTCAACGGCGG<br>CATGTTGATGCCTCCATGGCTGATATCGGATCCTGAATTCCAGCACACTGGCGGCCG<br>TTACTAGTGGNTCCNANCTCGGTACCAAGCTTGATGCATAGCTTGAGTANTNCTAAC<br>GCGTCNCCTAAATAGCTTGGNGTANCATGGNNATAGCTGTTTNC
3	ATGGCTCATAATATTTTTGTCACTGGTGCAACGTCTGGTATCGGTCTTTGTATTGCTG<br>AGGCTTATGCAAAGCATGGCGATAATGTGTTGATTTCTGGTCGTCGTGCTGAGTTAT<br>TGGGCGAGGTACAGGCTCGTTTGTCTAAGGAATATGGCGTGCGTGTTGAGACTTTA<br>GTTCTTGATGTGCGTAGTCGCGAGGATGTTGAAAGCAAGGTTCCTGCAGCTATCGA<br>GGCTTTTGGTGGCGTCGATGTGCTCGTTAATAATGCGGGTCTTGCACAAGGGCTTGA<br>TCCTTTCCAAGATAGTGCTGTTGATGATGCGGTGACTATGATTGATACCAATGTGAA<br>AGGCCTTTTATATGTAACAAAAGCAGTGCTACCTTTTATGATTGATAAAAATGAAG<br>GTCATATTGTAAATATGGGTTCTACTGCAGGCATTTATGCATATCCTAATGGCGCAG<br>TGTACTGTGCTACAAAGGCGGCGGTAAAGACATTAAGCGATGGCATTCGCATGGAT<br>ACCATAACTACAGATATTAAGGTTACTACCATTCAACCAGGCATCGTAGAAACTCC<br>ATTCAGTGAAGTACGCTTCCACGGTGATGCGGAACGAGCTAAATCTGTGTACGCTG<br>GTATTGATGCAATCCAACCAGAGGATGTGGCCGATGTTGTACTATATGTAACTAAC<br>CAACCTAAACGCTTGCAAATCTCTGATGTAACCATCATGGCGAACCAACAAGCGGC<br>AGGCTTTATGGTG*CCATGGCTGATATCGGATCCGAATTCGAGCTCCGTCGACAAGCTTG*<br>*CGGCCGCACTCGAGCACCACCACCACCACCAC*
4	ATGTCTAAAGTAGCTATTGTTACAGGTGCAGGTCAAGGAATCGGTTTTGCAATCGC<br>AAAACGCTTGGTCCAAGATGGTTTTAAGGTTGGAGTATTAGACTACAATGCTGAAA

CAGCCGAAAAAGCAGTTGCTGAATTGTCAGCAGACAAGGCTTTTGCTGTTGTGGCT
GATGTGTCTAAACAAGCAGAGGTTGCTGCAGCTTTCCAAAAAGTTGTAGACCATTT
CGGAGACTTAAACGTTGTGGTAAACAACGCTGGTGTCGCTCCGACTACACCTCTAG
ATACGATTACAGAAGAACAGTTCAATCTACCTTTGCTATTAACGTCGGTGGGGTAAT
CTGGGGAGCACAAGCTGCTCAAGCTCAATTCAAAGCACTTGGTCACGGTGGTAAAA
TCATCAATGCTACTTCTCAAGCAGGTGTTGTCGGAAATCCAAACTTGACTGTTTATG
GTGGTACAAAATTCGCTGTTCGTGGTATTACTCAAACATTGGCGCGTGATTTAGCTG
ACTCAGGCATCACTGTTAATGCGTATGCACCTGGTATCGTTAAGACTCCAATGATGT
ATGATATCGCTCACGAAGTTGGGAAAAATGCAGGAAAAGACGACGAATGGGGTAT
GCAAACATTCGCAAAAGATATTACATTGAAACGCCTATCTGAGCCAGAAGATGTCG
CTGCAGCGGTTAGCTTCCTTGCTGGACCAGATTCAAACTACATTACAGGTCAAACCA
TTATCGTCGATGGTGGTATGCAATTCCAT*CCATGGCTGATATCGGATCCGAATTCGAGC*
TCCGTCGACAAGCTTGCGGCCGCACTCGAGCACCACCACCACCACCAC

5 ATGTCAGAAACGATTTTAGTAACAGGAGCTTCAGCTGGTTTTGGTCAAGCGATTTGC
CGTCGCTTAGTAGCAGATGGATACCGTGTGATCGGATCAGCTAGACGCATCGATAA
ATTACAGGCACTTCAAGAAGAGCTGGGAGAAGCCTTTTATCCCCTGCAAATGGATG
TGACGGATCTTTCTCAGGTAGATCATGCACTTGCCAGTTTGCTAAAAGCTTGGGAGA
AAGTGGATGTTTTGGTCAATAATGCTGGCTTGGCTCTAGGCCTCGCCCCAGCTTATG
AAGCAGAGGTCGCAGACTGGCTGACCATGATTCAGACCAATATTGTCGGCTTGACC
TATCTGACAAGGAAAATCTTGCCTCAGATGGTGGAACGAAATGATGGCTATATTAT
CAATTTGGGTTCTACAGCAGGAACTGTGCCTTATCCAGGGGCCAATGTTTACGGGG
CATCCAAGGCTTTTGTCAAGCAATTCTCCCTCAATCTTCGGGCGGATCTAGCTGGCA
AGAAGATTCGTGTCAGCAATATTGAACCTGGTCTTTGCGAAGGGACAGAGTTCTCTT
CTGTTCGCTTTAAAGGAGACGAAAAACGGGTAGAAGCCCTCTATCGAGATGCCCAT
GCCATTCAGTCTGAAGACATTGCCAACACTGTAGCTTGGTTGATCCAACAGCCCAA
GCATGTCAATGTCAACCGGATTGAAATCATGCCGGTTTCCCAAACCTTTGGTCCTCA
ACCCGTTTTCGT*CCATGGCTGATATCGGATCCGAATTCGAGCTCCGTCGACAAGCTTGC*
GGCCGCACTCGAGCACCACCACCACCACCAC

6 ATGTACAGTGAATTAAAAGGAAAGGTAGCCGTCATTACAGGTGGATCTAAGGGAAT
CGGAACGGCTATCGCCAAGCGTTTCGGGCAAGAAGGCATGAAGGTTGTCATTAACT
ATAATTCCGATGCAGCCGGTGCAGAGTTGGCTGCAGAAGCGGTTCGATGTGCCGGC
GGAGAGGCGGTTACTGTCAAGGCTCATGTCGGTACGGAAGAAGGCGTGCAGTCTCT
GGTCGATGCGGCTGTAGAAAACTACAGCGGTATCGACGTATGGATTAACAATGCGG
GCATGGAAAACAAGGTGGCGACTCATGAAATGCCCTTAAGCGATTGGGAACGAGTT
ATTAACGTTAATTTAACAGGTGTATTTCTCGGTACACGTGCGGCTTTGACATATTTT
ATGGAGCATGATGTAAAGGGAAGTATTGTAAATATGTCATCAGTTCATGAGCAGAT
TCCTTGGCCGACCTTTGCGCATTACTGTGCATCCAAAGGCGGTATTAAACTCTTTAC
GCAAACTGTGGCCATGGAGTATGCCAAGTACGGAATTCGTGTAAATGCCGTCGGTC
CCGGAGCTATTAATACGCCTATTAATGCGAAAAAATTCTCCGACCCCGTTCAGTATG
AAGAAACTATGAGTATGGTTCCCATGAAGCGTATCGGTAAGCCTGAAGAAGTGGCA
GCTTGTGTGGCTTGGCTTGCTTCCGATGAAGCGTCATACGTAACAGGAATCACCCTT
TTTGTAGACGGCGGCATGACCCTGTATCCGGCTTTTCAAGACGGTAAGGGT*CCATGG*
CTGATATCGGATCCGAATTCGAGCTCCGTCGACAAGCTTGCGGCCGCACTCGAGCACCA
CCACCACCACCAC

7 ATGCCTCATAAAAATGATGTTCAAGTGGCATTGATCAGCGGTGGTACATCGGGAAT
TGGATTTGCCACAGCTAAGCTATTATTGCAAGAAGGTTGGTGCGTCGTTATCAATGG
CCGCGATGAACAAGCGGGACAAAGGGCAAAGATGAAATTGCGCCGATATTCTTCG
AAGGTGCGCTATGTGAAAGGCGATGTATCCAGTGTTAGCGATTGTCAGCGCATCGT
AAAAGAAACGGTAGATTTCTTTGGTAGTATTTCAGCTCTTGTAACGGCGGCTGGATA
TTATGAAGAAGAGCTACTAGCCGATGTATCTGAATCAGCCTTTGATGAAATGTTTGG
AACAAATGTAAAAGGAACTGTATTCTTGTGCCAAGCAGCATTGCCTTATTTACGTCA
AGTAAAGGGCAGTATTGTTACCGTATCTAGTGATGCAGGTCTACAGGGCAATGTAG
CCTGCTCAGTATATGGTGCGTCAAAAGGTGCTATCGTAAGCTTTACAAAATCACTGT
CCCTTGAGATGGCGCCCCATGAGGTACGCGTTAACTGCGTGTGCCCTGGAGATGTA

GATACATCGCTTGTAGATAAGCAAATTGCCCAATCCCATCAAGATGCGGAACAAGC
AAAAGAGGAGATGGGCCAACATTATCCGTTAGGACGTATTGCAAAACCTCATGAAA
TTGGCGAAGTTATCGCTTTTTTAATTAGTTCAAAAGCATCCTTTGTAACAGGTGCTG
CATGGACGATTGATGGAGGTCTTACGAGT*CCATGGCTGATATCGGATCCGAATTCGAG*
CTCCGTCGACAAGCTTGCGGCCGCACTCGAGCACCACCACCACCACCA

8 ATGACTAAACGCGTCTTAGTTACAGGTGTTAGCTCAGGGATTGGTCTTGCACAAGC
ACGTCTCTTTTTAGAAAATGGTTATCAAGTTTATGGGGTGGATCAGGGTGAAAAAC
CTGACTTGCAAGGTAACTTTCACTTTTTACAACGTGATTTAACACTAGATTTAGAGC
CTATTTTTGACTGGTGTCCTCAAGTTGATATTCTGTGTAATACTGCAGGAATTTTGG
ATGACTACAAATCTTTACTGGAACAATCAGCCCAAGAAATCCAAGAGATTTTTGAA
ATTAACTATGTGACTCCAGTAGAGTTGACTCGCTATTATTTGACTCAAATGTTGGAA
TACAAGCGAGGAACCATCATCAATATGTGTTCCATTGCTTCAAGTCTGGCAGGCGG
AGGTGGGCACGCCTATACCTCTTCTAAACATGCCTTAGCAGGCTTTACCAAGCAATT
AGCTCTAGACTATGCTGAAGCTGGGATTCAGGTCTTTGGCATTGCTCCTGGTGCGGT
TAAGACTGGTATGACAGCAGCTGACTTTGAACCAGGTGGTCTGGCAGACTGGGTAG
CCAGTGAAACACCTATCAAACGCTGGATTGAGCCAGAAGAAGTGGCAGAAGTCAG
CCTCTTTTTAGCAAGTGGAAAAGCAAGCGCCATGCAAGGACAAATCTTGACTATTG
ATGGTGGCTGGTCTTTG*CCATGGCTGATATCGGATCCGAATTCGAGCTCCGTCGACAAG*
CTTGCGGCCGCACTCGAGCACCACCACCACCACCAC

9 ATGAGCTGGGTCATTGTGACCGGCGCGAACGGCGGTATTGGTGAGGCAACCGTCCA
CCAGCTCATCAAGAACGGCTATTCGGTGTTTGCGGCTGACCTTGCGGAGCAGCCCA
TTGCCTCCTTCGGTACCTACGGGGACGCAATCTTCCGCTACCGCGCCGTAGACGTTA
CGAGCGAAGAGTCCGTCACCGCCCTCGCAGAAGCCGTTGCGGCGCTTGACGAACCC
ATCACCGGCGCGGTGCTCGCCGCGGGTATCGCCCACAGTCAGCCGCTGCTGGAAAC
CAGCTTCGCCACCTGGAAGCGCCTGCACGCGGTCAACTCCGACGGCGTGTTCCTGT
GCCTGCGCGAATTCGCCCGCATCATGATTGACCAGCAGGAGTCCGACCCGACCAAC
AGCCGCTCCCTGGTGACGGTCGCCTCCAACGCGGCGCGTGTGCCCCGCGCGGAGTT
CGGCGCCTACGGTGCGTCCAAGGCGTCGGCGGCACGCGTGAGTTCCAGCTTCGGTC
TGCAGCTTGCCGCGCACGGTATTCGCGTGAACTCGGTCTGCCCCGGCACCACCCGA
ACCCCCATGGTGACCGACGCCTGGGAGGGCGAGGACCGCTCCGCCCTGCCGGTTGC
CGGTAACCCGCAGACGTTCCGCCTGGGTATTCCGCTGGGTCGCATCGCCGACCCTGC
CGACATCGCGGCGGTCAACGCCTTCCTCATTTCTGAGGCGGCACGCCACATCACCAT
GCAGGAGATTGTCGCC*CCATGGCTGATATCGGATCCGAATTCGAGCTCCGTCGACAAGC*
TTGCGGCCGCACTCGAGCACCACCACCACCACCA

10 ATGACTGAGAACACCCACATTCCTGCATCTCCTTTCCTGCCCCATACCGGTGACGGC
AAAGTTGCCGTGGTGACCGGCGCATCCAGCGGTATTGGCCGAGCCACCGTACAGCA
GCTAGTAGCAAGTGGCTGGACCGTCTATGCTCTGGCTCGTCGTACCGACCGTCTTTA
CACCCTTTATGCGGAGACCGGCGCTCATCCGGTGACCTGCGATGTGACCGATGAGC
AGTCCGTTCAGTTCGTAGCCGAGCAGATCCTGGAAGAGCAGGGCACCATTGATGCC
CTGGTCAATATTGCTGGCGGTGCTATCGGTGTGGACAAGGTTGCAGAGGGTAAGCC
TGATGACTATCTGAAGATGTATCAGATGAATGTTCTTGGCATTCTGCATATGGTGCG
TGCCTTCGCGGAGGCGCTGCGCCAGAATGGTTACGGTACGATTCTGAACCTGACCT
CCACCGCTGCCGAGCACGGCTATGAGGGAGGCGCCGGGTACAACGCGGCGAAGTT
CGGCGCTCGCGGCCTGACCGAGGCGCTTCGCCTGGAGGAGGCGGAGAATAATATTC
GCGTGATTGAGATTTGCCCGGGCATGGTGCACACCGAAGAGTTCTCTCTAAACCGC
CTGGGGTCCAAGGAAGCAGCCGAGCGCGTCTACGCGGGCGTTGAGAAGCCGCTGA
CCGCAGAGGACGTGGCGCAGACCGTGACCTTTGCGTTGAACGTTCCGCACCATGTG
AACCTTGACCGTATTACGATTCGCCCGGTGGCCCAGCCGAGTCAGTTCAAGGTGATT
CGCAAGGAGGGC*CCATGGCTGATATCGGATCCGAATTCG*
AGCTCCGTCGACAAGCTTGCGGCCGCACTCGAGCACCACCACCACCACCAC

11 ATGCCCAAGTCACTGCAACGCCCCGTCGTTCTTCTCACCGGCGCCAGCTCCGGTATC
GGCTACGACGTGGCGCCGCTACTGGTGCGCTACGGCTACATCGTCTACGGTGCCGC

CCGCCGCGTTGAGAAGATTGAAGAGCTCGCCTCCGAGGGCGTGAAAGCGCTCAGCC
TGGACGTTACCGATGAAGCATCCATGGAAGCCGCCGTACAGCAGATCATTGATGCA
GAAGGCCGCATTGACGTGCTCATCAACAACGCTGGCTACGGATCCTACGGCGCTAT
TGAAGACGTCCCCATCGACGAGGCGCGCCGCCAGTTCGAGGTGAACCTCTTCGGCC
TGGCGCGCCTCACCCAGCTGGTACTGCCGCATATGCGCGCACGGGGCAGCGGGCGC
ATCCTGAATATCTCCTCAATGGCGGGGCGTATTACCTCGCCCCTGGGTGCCTGGTAC
CACGCCACCAAGTATGCCCTCGAGGCGTTCAGTGATGCACTGCGCATGGAGGTTGA
AGAGTTCGGTATTGACGTGGTCATTATCGAGCCCGGCGGCATCAAAACCCCCTGGG
GACTCATCGCGGCAGACCACCTCGAGGAATCCTCCCGAAACGGTGTCTACGCGGCT
CAGGCGCAGCGGGTTGCGGCAAATATGCGTAGGCTCTACTCTCCTTCATCCAATCTC
TCGGAGCCCAAGGTTATCTCTAACGCTATTCTGCGAGCCCTGGAGGCGCGCCGCCC
CAAGACCCGCTACCTGGTAGGTTTCGGTGCTAAGCCCTCCGTGTTCCTGCATACCGT
GCTACCGGACCGTCTCTTTGACAAGGTGGCGCGCCGTATCTTC*CCATGGCTGATATCG*
GATCCGAATTCGAGCTCCGTCGACAAGCTTGCGGCCGCACTCGAGCACCACCACCACCA
CCAC

12 ATGAGTAACGCTCCCGAGGTACGTGGGCTTTTCCTCAAGGCCCTCGGCCGCCCGGTC
ATCGTGGCCCCCAGCTCGGCGGAGCCGACAGTCACCTTCGACGGTCCGCTCACCGA
GGTGTGCTCCTGCTCCCTGAAGGAGACGGAGCTTCCCGTCGTCGTGCGCGCAGGCG
AGGAGACTTTCGAGGTTCGCGCCACTTCCACCGGCGAGCGCACCATCAACGGCCGC
GTCGCCCTCGTGACGGGCGGCGCCCAGGGTTTGGCGCGGAGATCGCGCGCGGCCT
GGTCGACGCCGGTTGCTTCGTGTTCATCGCCGACCTGAACGGTGAGGGTGCCGCCG
CTAAGGCGGCCGAGCTCGGCGGCGAAGGCGTCGCACACCCGATCACGGTTAACGTC
GCGGACGAGGAGTCCGTCGCGGCGATGGCCGCCGAGATCGAGCGCGTCACCGGCG
GCCTCGACCTGGTCGTGTCCAATGCGGGTATTGTGCGTGCCGGCTCCGTCCTCGAAC
AGGACGCTTCCGCGTTCCGCCTGTCCACGGACATCAACTACGTGGCCTTCTTCCTGG
TCACCAAGCACCTGGGCCAGCTTCTGGCCCGCCAGCACTCGACCGCCCCCGAGTGG
CTGACCGACATCATCCAGATCAACTCCAAGTCTGGCCTCGTCGGCTCGAACAAGAA
CGCCGCCTACGCGGGATCGAAGTTCGGCGGCATCGGCCTAGTGCAGTCCTTCGCCC
TCGAGATGGTTGCTCACGGCGTGAAGGTCA
ACGCGATCTGCCCCGGTAACTTCTACGACGGCCCGCTCTGGTCCGACCCGGATCGC
GGCCTCTTTGTCCAGTACCTGAACTCCGGCAAGGTCCCCGGCGCGAAGACGGTCGC
CGACGTCAAGGAGTTCTACGAGGCGAAGGTCCCCATGCGCCGCGGCGCTCAGGGCA
TCGACGTTCTGCGCGCGATCTTCTACATCGTCGAGCAGGAATACGAGACCGGCCAG
GCCGTGCCCGTCACGGGCGGCCAGGTGATGCTCTCCCATGG

13 ATGAACACTTCTCGTCGCGTCGTTGTCACTGGAGCCTCCACCGGAATCGGACAGGC
CACCGCCCGCCTGTTGGCAAAGCGGGGGTGGAAGGTCGTCGCCGTGGCCCGGCGCC
GCGAACGCCTCGAGGCCCTCGCCGAGCAGATTGGCTGTGAGTACTGGGCGGCCGAC
CTCACCGATGAGGCCCAGGTGAAGGAGATGGCCGCCCACGTCCTGGAGGGGGGTCC
CGTGGATGCGGTTGTTAACAACGCCGGGGGAGCGATCGGCGTCGACCGTGTTGCCG
AGGGTGATCCCGCCCGGTGGAGCGCGATGTTCGAGCGCAACGTGCTCACCGCCCTG
CACTGCTCGCGCGCGTTCTTGCCGGGGATGCGCGAGCGCGGGGGAGACCTCGTGTT
CCTGACCTCGACCGCCGCCCACGACACCTACCCGGGTGGCGGCGGCTACGTGGCCG
CTAAGCACGCCGAGCGCATCATCGCCAACACGCTGCGTCAGGAGCTGGTGGGCGAG
CCTGTGCGCGTCATCGAGATTGCGCCCGGCATGGTGCGTACCGAAGAGTTCTCGCTC
AACCGGCTCGGCTCCCAGGAGGCGGCCGACCGCGTCTACGAGGGGGTCGCCGCCCC
GCTCGTCGCCGAGGACATTGCGGAGGCGATCGTGTGGACCCTGGAGCGCCCCTCCC
ACGTCAACATTGACTCGATGATCGTGCGCCCGGTGGCCCAGGCGACAAACACGCTC
GTGGCCAGGAAGACCGCC*CCATGGCTGATATCGGATCCGAATTCGAGCTCCGTCGACA*
AGCTTGCGGCCGCACTCGAGCACCACCACCACCACCA

15 ATGGACATTGCAGATGCCGAGGGCGTCAAGAAGACCGTTGCTCAGATTCTTGAGGA
GTGCGGCGACGTCAACGTTCTTATCAACTGCGCTGGTCGCATCAGCTCCGTTCCTTT
CACCGAGGTTGACGACAAAGAGTGGAACAACACTATCAACACCAACCTCACCGGTA
CCTACAACGTAACTCACGCTCTCTGGCAGCACTTCATTGATCGTGGTGGCGCTCGCA

	TTGTCAAC<br>GTTTCTTCCGTTGCAGGCAAGATTGGCGGCGGCCTCCTTGGTACTGTTGCTTACGCA<br>TCTTCTAAGGCTGGTATGAACGGCTTTACCAAGGCTATCGCTAAAGAGGGCGGCAA<br>GTACGGCATTTCTTGTAACGCTGTTTGCCCATCCTTCACCATTACAGATATGACCAC<br>TGCACTCTCCAACGATGAGGAGAAGTACAAGAAGGTCGTAGGCATTATTCCTCTCG<br>GCCGTCCTGCACAGGCATCTGAGCCTGCACAGATGGTACTGTTCTTTGCTTCCGACG<br>CTGCTAGCTTTGTCAACGGTGAGGTTGGCGACTGCGACGGCGGCATCGTGATGGAA<br>*CCATGGCTGATATCGGATCCGAATTCGAGCTCCGTCGACAAGCTTGCGGCCGCACTCGA*<br>*GCACCACCACCACCACCA*
16	ATGAGCGAGAACACTCCCAAGACCGTGCTGGTCACCGGCGGCAACCGAGGCATCG<br>GCTACGAAATTGCCAAGGAATTCCAGGCAGCAGGACACAACGTCTGCATCACCTAC<br>CGTAGCGGTGAAGCCCCCGAAGAATTTTTCGCCGTCAAGGCAGATGTACGCGATGC<br>CGACAGCATTAACGAAGCATTCAAGGAAATTGAAGCAGAGTTCGGCCCCGTGGAG<br>GTGCTGGTCGCCAATGCAGGTATCACCCGCGACATGCTGCTGATGCGCATGAAAGA<br>ATCCGACTTCACCGACGTGGTCGACACCAACCTCACCGGTTCTTTCCGCGTGGTTCA<br>GCGCGCCATCAAGGGCATGCTGAAGCTCAAGCGCGGTCGTATCATTCTGGTTTCCTC<br>CGTGGTGGGTCTGTACGGTTCTCCCGGCCAGGTCAACTACTCCGCGTCCAAGGCCGC<br>ACTGGTCGGTATGGCGCGCTCCATCACCCGTGAGCTGGGCGGTCGTAACATCACCG<br>CTAACGTGGTGGCACCGGGCTTCATTAACACCGCAATGACCGAGGTTCTGCCCGAA<br>GAGACCAAGAAGAACTACCTGGCGTCCATCCCGGCTGGCCGTTTCGCTGANGCGGA<br>AGAGGTCGCTCGCGTGATTCGCTGGCTCGCATCCGATGAGGCTAGCTACATTTCTGG<br>CGCTGTCATCCCCGTTGATGGCGGCCTGGGCATGGGTCAC*CCATGGCTGATATCGGAT*<br>*CCGAATTCGAGCTCCGTCGACAAGCTTGCGGCCGCACTCGAGCACCACCACCACCACCA*<br>*C*
17	ATGGAACTTAAAAATAAAAATGTTTTTGTAACAGGTTCAACACGCGGAATTGGATT<br>GGCTGTGGCTCATAAATTTGCGAGTCTCGGTGCCAATGTTGTCCTAAATGGACGTTC<br>TGAAATTTCTGAGGACTTGCTTGCACAGTTTGCTGACTATGGTGTGACTGTTGTTGG<br>TATTTCTGGGGATATTTCTAATGGCGAAGATGCGCAACGTATGGTAGCTGAAGCAA<br>TTGAAAAGCTTGGAAGTGTTGATGTTTTGGTCAATAACGCTGGCATTACAAACGAC<br>AAGTTGATGTTGAAAATGACTGAAGAAGATTTTGAACGGGTCTTGAAAATCAACTT<br>GACCGGTGCCTTCAATATGACTCAAGCTGTCTTAAAACCTATGTCTAAGGCTCGTCA<br>AGGTGCCATTATCAACATGTCCTCTGTAGTAGGTCTTATGGGGAATATCGGTCAAGC<br>AAACTATGCAGCTTCAAAGGCTGGTTTGATTGGGTTTACCAAATCCGTTGCGCGTGA<br>AGTTGCGGCCCGTGGTGTTCGTTGAATGCCATTGCACCTGGTTTCATTGAATCAGAT<br>ATGACAGACGCTATTCCAGAGAAAATGAAAGATGCCATGCTAGCTCAAGTGCCAAT<br>GAAACGAATTGGTCAAGCTGAAGAAGTGGCAGAAGTTGCGGCTTTCTTAGCAGGTC<br>AAGAATATTTAACTGGTCAAACAATTGCCATTGATGGCGGAATGACTATGCAA*CCA*<br>*TGGCTGATATCGGATCCGAATTCGAGCTCCGTCGACAAGCTTGCG*<br>*GCCGCACTCGAGCACCACCACCACCACCA*
18	ATGGCAATTTTGATTACCGGTGCATCGGCAGGATTCGGCGCGGCAATGTGCCGTAC<br>TTTTGTTGCGGCAGGCTATCATGTCATTGGCGCGGCACGGCGCGAAGACAAATTGC<br>AGCAGTTGGCAGAGGAATTGGGCGAGCAGTTTTACCCTTTGGAAATGGATGTTTCG<br>CGCACGGAGTCGATTCAAAACGCTTTGAACAGCCTGCCCGAGCATTTGTCCGAAAT<br>CGATTGCCTGATCAACAATGCAGGTTTGGCTTTGGGTTTGGATACTGCCGATAAAGC<br>CGATTTTGGCGATTGGGAAACCATGATTCAAACCAATATCATCGGCCTGACTTTTCT<br>AACCCGCCAGATTTTGCCGCAGATGGTAGCGCGCAAACAGGGCTATATCATCAATT<br>TGGGTTCGATTGCCGGTAGTTATGCTTATTCCGGCAGTAATGTCTATGGCGCGACTA<br>AGGCTTTTGTGCGTCAATTCAGTATGAACCTGCGCGCCGAATTGGCAGATAAAAAT<br>ATCCGCATTACCAATATCGAACCGGGTTTGTGCGGCGATACCGAGTTTTCCAATGTC<br>CGTTTCAAAGGCGACGACCAGCGTGCGGCCGAAGTTTATGAGAATGTCGAGTTTAT<br>CCAGCCGCAAGATATTGCTGATACCGCTTTGTGGCTCTATCAGCGTCCGGCGCGGAT<br>GAACGTCAATTCCATCGAAATCATGCCGGTTGCCCAGACCTTTGCAGGCATGAAAG<br>TTTACCGCGACGAGCCTGCCCCGGCGAAAGAAGAAACGTTTGAAAAACAAAGTATG

	TCTTTGTTTGGAAAAATTAAATCTTGGTTTAAG*CCATGGCTGATATCGGATCCNAATTC*<br>*GAGCTCCGTCGACAAGCTTGCGGCCGCACTCGAGCANCACCACCACCACCAC*
19	ATGAATGATTGGTTAAACATAAAAGGCAAAACAGTTCTTGTGACTGGCGCGTCATC<br>TGGGATCGGTAAGGCAATCGTTGAAGAATTGCTGGAATTAGGTGTCAATGTAGCGA<br>ATTTCGATCTTAGCGACAACGATTTGCGTCATCCAAATCTATTATTTGTAAAAGTTG<br>ATGTAACTTCTCGCTCTGAAGTAGAAGAGGGTGTTGCCAAAATAGTTGAAAGATTT<br>GGCAATATTGATGCAGTGGTCAATAATGCAGGGATTAATATCCCTAGATTATTAATC<br>GATGCAGAAAATCCTAAAGGTCCTTACGAATTGGACGATGAGACGTTCGAAAAAGT<br>AACGATGATTAATCAAAAAGGTTTGTATCTGGTTAGTCAGGCAGTGGGACGTATTTT<br>AGTGAAAAATGGAAAAGGTGTAATTGTGAACATGGCTTCAGAAGCAGGCTTGGAA<br>GGTTCTGAAGGACAAAGTGCCTATGCTGCCACAAAAGCAGCAGTCTATAGTTACAC<br>TCGTTCATGGGCAAAAGAGCTGGGTAAGCATGGTGTACGTGTGGTTGGAATTGCTC<br>CAGGAATTATGGAAGCTACGGGACTTCGAACTCTTTCTTATGAGGAAGCTCTTGCTT<br>ATACCCGTGGAAAGACGGTAGAAGATATTCGAGCTGGTTATGCTTCAACTTCAACA<br>ACTCCTTTGGGACGAAGTGGTAAACTACGGGAAGTAGCTGACCTTGTAGCATTCTA<br>TATTTCAGACCGTTCTAGCTATATAACTGGAGTCACTACAAATATTGCCGGANGGA<br>AAACTCGCGGT*CCATGGCTGATATCGGATCCGAATTCGAGCTCCGTCGACAAGCTTGCG*<br>*GCCGCACTCGAGCACCACCACCACCACCA*
20	ATGTATCTTTGCAAGCAAATATATCACACTGATAAACATAGAAAAACAAATATGGC<br>AAAAGTATTGGTAACAGGTGCTAACAAAGGCATTGGCTATGGCATCTGTAAGTTTT<br>TAGGCAAGAGTGGCTGGCAGATAATCGTTGGTGCGCGTAATAGTGAGCGTGCCGAG<br>GAAGCGATGAAGTCATTGAAAGCTGAGGGAGTGGACGTGATTGGATGGCAATACG<br>TCAATCTATCAGACAATGCTTCTTTGGAGCAAACAGCAAAAGAAGTAAAAGAGAAA<br>TATCATGACTTGGAACTATTGGTAAACAATGCTGGTATTCCTGGAGATATGGAAGT<br>AGCAAGTTATGAGTCAGAGCTAAAGGATGTAATAGATACCGTACAAGTAAACTATG<br>TCGGAACATTCTGCTTGACAAAGGCACTTACCCCATTACTCTCTGCAAACAAAGGA<br>AGAATTGTGAACATAACCGTACCATCTGAAGTAAGTCCTTACTGGCATCCAATGGC<br>TTACGTAGCCAGCAAAGCAGCA<br>CAGAATGCAATGACCAGCATTATGGCTATGGAATTTGAGAAGAACAACATACCTGT<br>TGAAATCTTCAATATCCACCCTGGAGCTACGACAACCGATTTAAATAACCATTATAC<br>AGGACCAGGTTCTCACTCGATAGATGTTGTTAGTGAGAAGATTGCAGAGGTTATTA<br>ATGATGGGGGAAAGCATCAAGGAGAGTTTGTTGAACTATATCCTATC*CCATGGCTGA*<br>*TATCGGATCCGAATTCGAGCTCCGTCGACAAGCTTGCGGCCGCACTCGAGCACCACCAC*<br>*CACCACCAC*
21	ATGAAATTATTGGAAGGTAAGACAGCTCTGATTACAGGTGCTGCACGTGGTATTGG<br>TAAAGCAATCGCATTAAAGTTTGCAGAAGAGGGTGCAAACATTGCATTTACCGACC<br>TTGTTATCGATGAGAATGGTAAAGCAACAGAGGCAGAGATTGCTGCTAAGGGCGTA<br>AAAGTAAAGGGTTACGCAAGTAATGCTGCAGATTTCGCACAGTCAGAAGAGGTTGT<br>AAAGCAGGTTAAAGAGGAATTTGGTTCTATTGATATTCTTGTTAACAATGCAGGTAT<br>AACAAAAGATGGTCTTATGCTTCGTATGACTGAGCAGCAGTGGGATGCTGTTATAG<br>CAGTGAACCTTAAGAGTGCTTTCAACTTCATTCATGCTTGTGTTCCAGTAATGATGC<br>GTCAGCGTAATGGTAGTATTATCAATATGGCCAGTGTAGTCGGCGTACATGGTAAT<br>GGTGGTCAGGCTAATTATGCTGCATCAAAGGCTGGTATGATAGCCTTGGCTAAGAG<br>TGTTGCACAGGAGATGGGCCCTAAGGGTATCCGTGCAAATGCTATCGCTCCTGGTTT<br>CATTGATACTGCAATGACACAGGCTTTGAACGACGACATTCGTAAGGAATGGACAT<br>CAAAGATCCCTCTCCGCCGTGGTGGTACTGTTGACGATATTGCTAACACAGCAGTAT<br>ATCTTGGCTCTGAACTATCAAGTTATGTGTCTGGTCAGGTAATCCAGGTTGATGGCG<br>GTATGAATATG*CCATGGCTGATATCGGATCCGAATTCGAGCTCCGTCGACAAGCTTGCG*<br>*GCCGCACTCGAGCACCACCACCACCACCAC*
22	ATGATGACGTCTCTCAGAAAAAAGATAATAGCTATTGGAGGTGCATTGGCTTACCC<br>ATCCATAAGTCTAACACCTGATATTACAAGAGAACACTTCTCTGGGAAGTGGGTTCT<br>TGTGACGGGTGCATCGCATGGAATAGGGAGAGCTTTAACCGAAAAAATAATCAATG

CAGGTGCAAATGTCTTTCTCATTGCTCGAAGCGAAGCTGACTTACGCCTCTTATGTG
CCAAAGCTAAGCAAATGGGTAGCAGTGCAGACTACTGCGCAATAGACCTAAGAGA
CAGGGAAAAGTTGGAACAGCTTTGCCAAAAACTAAGGGAAACACTACCACGGTTA
GATTACTTCTTTTGCAATGCCGGTAAATCTATCCATCGCAAGATCAATGATGCGCAG
GACCGCCTACACGACTATGACCGTACGATGGACCTCAACTATCGTTCCTTGGTTGCG
CTATCATTAGCTATCTTACCTGCCTTGAAAGCAAGTAAAGGGAGTATCATCTATTCT
TCATCAGTGAGTACACTCTATCCAATGGCACCGGGCTGGTCGGCTTATCATGCCTCA
AAAAGTGCAGCAAACACATGGTGCGAGACGGCAAACAGCGAGTTTGCGCCGTTAG
GTGTTCACGTGCAGATAGCTTATTTACCCTTGGTTCACACTGCGATGTCGGATGTCA
ACGAACAATACAAGCACTTACCTGCATACACACCAGCTGATGCAGCCAATATTCTA
TTGAAACTTGCTATACGGAAGGTCAGGACTTATAAACCTTGGTGGGCAAAGTTATC
AGCCCCAATAGCCTATCTCTTTGCTCCGATTATTCACCTATATTATAAGCGA*CCATG*
GCTGATATCGGATCCGAATTCGAGCTCCGTCGACAAGCTTGCGGCCGCACTCGAG
CACCACCACCACCACCAC

23 ATGAAAAAAGCAATCGTCGTAGGCGCCAGCAGCGGTATCGGACATGAAGTAGCAC
GACTGCTCATCGCACAAGGATGGGCGGTTGGTGTGGCTGCTCGTCGTATAGACAAG
TTGACAGACTTGCAAGCTATGGCACCAGAGCGGGTCTACACCGCCCAAATTGATGT
AAATAACGAAGATGCAGAGACTTCCTTACTGCAACTTATAGAACGTATGAACGGCA
TCGACCTCTATTTTCATGCCGCAGGAATCGGTTGGCAAAACCCAAGTCTTAATGCTG
ACATAGAACTCAAAACAATGGAAACCAATGCTGTTGGATTCACACGAATGATTGGC
TGTGCCTATCGCTATTTTGCCAATAAGGGAGGTGGACACATCGCTTGTATCACTTCT
ATCGCAGGAACAAAAGGGCTCGGACCGGCTCCTGCTTATAGTGCAACAAAGGCGAT
GCAGAACACCTATCTACAAGCGTTGGAACAACTCGCAGCTTGTAAACATCATAACA
TCCACTTCACAGATATTCGTCCCGGCTTTGTTGACACTCCCCTACTCGCTGGTACATC
TCACCTTCCGATGCTGATGACTACAGAAAAGGTGGCACGCAGTATTATAAAGGCTA
TTAACAGCCGACGACACATCTGCGTCATCGATAGCCGTTGGTGCGTACTTACCTACT
TATGGCGACATATCCCTAACTGGATATGGAGGCGAATGAAGCTATGTCAA*CCATGG*
CTGATATCGGATCCGAATTCGAGCTCCGTCGACAAGCTTGCGGCCGCACTCGAGCACCA
CCACCACCACCAC

24 ATGAAGATGGAAGATACAATTAAAAAAGGGATCATTATAGGAGCTACAGGAGGGA
TTGGTCGTCAATTAGCGAAAGAATTGGCACAACGTTTGGAGCATCTGATTTTAGTGA
GTCGAGATGCTGACAAACTCTCCCAAGTCCAAAAGGAACTAACTGGGAGTAAGGCG
CAACTTTCAATCCTAACATTAGATATGCTAGATCAAGTGGCTCTAGAAGCTTTTGTA
GAGAGTCTAGATGCTGATCTTCTTGTTAATTGTGCAGGTTTAGCTTACTTTTCCCGTG
AAAGTGACCTTGATTCAGCCAGTGAGCAAGACCTCTGGCAAGTCAACTACCATAGT
TCAGTCCAGCTAATCAAGCAGGTGGTGCAAAAGAATCAGAAGATCCAGCTGGTCCA
GCTGTCTTCGCTGGCAGCTCTTTTTCCTCATCCCTATCTAGCAGCCTACAGTGCTAGC
AAGGCTGCCTTGCAGACCTTCACTCTTGCTCTCCAGGAGGAACTGAGGCAATCAGA
ATCCCAGGTCCAGCTAGGTCTCTATATTCTTGGACCAGTCCAAACAGCTATTTTTCC
TCCCAAACTAGTAGAAGCATTGGGTGGCAGTCGCTTGCAGATGAAGTCTGAGAAAG
TCGCTCAGCAGTTGATTCGATTCATAGAAAGGGATACTTCCTATGAGATTATCGGGC
TTCGCTATCGCTTGCTAGTTTGGCTAGGTCGCCTGCTTCCCCAAAGGTGGATCATCC
GTCTCCTTGCTATATATTTAAAAAAAGGGACT*CCATGGCTGATATCGGATCCGAATTC*
GAGCTCCGTCGACAAGCTTGCGGCCGCACTC GAGCACCACCACCACCACCAC

25 ATGAGTTATAACTTATTAAAAGGTAAAAGAGGCATTATTTTCGGTGCACTCAACGA
GCAGTCAATTGCATGGAAAGTTGCAGAACGAGCTGTAGAAGAAGGAGCATCAATA
ACACTTTCCAACACTCCTATAGCCGTTAGAATGGGTACTGTTAATTCATTAGCAGAG
AAACTGAATTGCGAAGTAATTGCAGCTGATGCTACAAGTGTAGAAGATTTAGAGAA
TGTATTTAAGCGTTCAATGGAAATTCTAGGTGGAAAAATTGACTTTGTACTCCACTC
TATTGGTATGTCTCCAAACGTTCGCAAACATCGCACATACGACGATCTGGATTATAA
TATGTTGAATACAACGCTTGACATATCGGCTGTATCATTTCATAAAATGATTCAGAG
TGCTAAGAAACTTGATGCCATAAGCGACTATGGTTCTATTCTAGCTCTATCGTACAT
CGCTGCGCAACGTACATTCTTTGGTTACAACGATATGGCAGACGCAAAAGCATTAC
TAGAAAGTATTGCACGTAGCTTCGGTTACATATACGGTCGCGAGAAGAATGTACGT

ATCAACACAATATCACAATCTCCTACAATGACTACTGCTGGTCAGGGCGTAAAGGG
AATGGATAAATTGTACGACTTTGCTAACCGTATGGCGCCACTTGGTAATGCTTCTGC
TGCCGAATGTGCAGACTACTGTATTGTTATGTTCTCAGACTTAACAAAGAAAGTTAC
AATGCAAAATCTCTATCACGATGGCGGTTTCTCTAACATTGGTATGAGTCTTCGTGC
AATGACAACCTATGAAAAAGGTATTGGAGACGAATATAAAGATGAGAACGGAAAG
ATTATCTACGGA*CCATGGCTGATATCGGATCCGAATTCGAGCTCCGTCGACAAG*
CTTGCGGCCGCACTCGAGCACCACCACCACCACCAC

26 ATGAGTGAAACTATTTTAATTACAGGGTCTAATCGCGGTATCGGCAAAGCCGTCGC
GCTTGGTTTAGCGCAGGACGGCTTTGATATTGTTGTCCACTGCCGTAGCCGCCGTGA
TGAAGCGGAAGCTGTCGCGGAAGAAATCCGTGCATTAGGTAGAAATGCACGCGTGT
TGCAGTTTGACGTGTCCGACCGCGAAGCCTGCCGAGAAATTTTGACTGCCGACATT
GAGGCAAACGGCACATATTACGGCGTGGTGCTGAATGCCGGCCTGACGCGCGACAA
TGCGTTTCCTGCGTTTACAGATGATGATTGGGACTTGGTGCTGCGTACCAATTTGGA
CGGTTTTTATAATGTGTTGCACCCTTTGACCATGCCGATGATTCGCCGCCGCAAAGC
CGGACGGATTGTGTGCATGGCGTCAGTATCCGGTTTGACGGGCAACCGCGGTCAGG
TCAATTACAGTGCGTCCAAAGCGGGTCTGATCGGCGCGGCAAAAGCCTTGGCGGTT
GAGTTGGCAAAACGAAAAATTACCGTCAACTGTGTGGCACCGGGTCTAATCGATAC
CGATATTATCGATGAGAACGTACCTGTCGAAGAAATCTTAAAGGCCGTCCCTGCCG
CGCGTATGGGATTGCCGGAAGAAGTGGCGCATGCGGTGCGCTTTTTGATGGATGAG
AAAGCGGCGTACATTACGCGCCAAGTGATTGCTGTGAACGGAGGTTTGTGT*CCATG*
GCTGATATCGGATCCGAATTCGAGCTCCGTCGACAAGCTTGCGGCCGCACTCGAGCACC
ACCACCACCACCAC

27 ATGGCTTATAACTTATTAAAAGGAAAACGTGGTGTTATTTTCGGTGCACTGAATGATA
TGTCAATTGCATGGAAAGTGGCTGAACGCTGCGCAGAAGAAGGTGCAACTCTCGTGT
TGAGTAACACTGAAATGGCACTTCGTATGGGTTCACTTGATGAACTTTCCAAGAAGA
TTAATGCGCCAGTTATTGCGGCAGATGCGACCAATTACGATGATCTTGAGAATGTTTT
TGTTAAGGCTCAAGAGCTATTGGGAGGCAAAATAGACTTCGTTCTCCATTCCATAGG
GATGAGTCCTAACGTTCGTAAGCACCGTACTTACGATGATCTTGACTACAATTGGCTA
AATAAAACGCTTGATATTTCAGCCATTTCATTCCACAAGATGCTGCAAGCTGCAAAG
AAAGTTGACGCAATTGCAGAATATGGTTCAGTCGTTGCATTGACGTATGTGGCTTCAC
ATCGCACATTCTTTGGCTATAACGACATGGCCGATGCAAAGAGTTTATTGGAAAGTA
TAGCACGTAGTTTCGGCTATATCTATGGCAGAGAGAAGAATGTTCGCATTAATACCA
TCTCACAATCTCCTACAGAAACAACTGCAGGTAAAGGCATTAAGGACATTGATAACA
TGATGGATTTTGCTGATAAGATGTCACCTCTCGGCAACGCTACAGCCGATGAGTGTG
CTGATTACTGTGTAACTTTGTTCAGTGATCTAACCCGTAAGGTGACAATGCAGACCCT
TTTCCACGATGGAGGCTTCTCAAACATGGGTATGAGTCTGCGTGCTATGAACCAATAT
AGTAAGACTTTGGAT*CCATGGCTGATATCGGA*
TCCGAATTCGAGCTCCGTCGACAAGCTTGCGGCCGCACTCGAGCACCACCACCACCACCA

28 ATGAACATTGCTATTATTACCGGTGCATCCCGAGGAATCGGTAAAGCCGCGGCCAAG
CGTTTTGCAAGAGAAGGTTACAGCCTCTTATTAAATTGCGAAAAAAACTGGACGCTT
TTGGAAGAATTGAAAAAGGAAATTCAGTCAGACTTGCCGGAAAACTGTCCGGAAATT
TTTCTTTGCAAAGATTTGGGCACAAAAAAAGGCCTTTCCCGTATTCTTGAAGGAAAA
AGCCTATCTAAGCTAATTCTTATCGCCAACAGCGGTAAAGACGCTATCAAATTATTGC
AAGACTGCAGGGAGGAAGAAACAAAGGCTCTTTTGGAAACAAATCTTTTACAGCCCT
TTATTCTTTGCCAAAAGCTTCTTCCCTATCTTTTACAAGCTGAGGAAGGGAGAATTCT
ATTCAGTTCCTCTGTCTGGGGAAATGTGGGGGCTTCCATGGAAAGTCTTTATTCCCTC
ACCAAGGGCGGTATCTCCACCTTTGCGAAAGCTCTGGGAAAAGAGCTCGCCCCTTCT
CACATCTCCGTTAATGCCGTAGCTTTCGGTGCTGTTGACACGGATATGAATTCCTGGC
TTAGCACCGAAGAAAAGCAAAGCTTAGAAGAGGGCATCCCCTACGGCAGAATGGCT
ACCGTAGAAGAAGCCGCAGACTTTCTTTACCTTCTCTCCCAAGCCCCCCTCTACCTTA
CAGCCCAAGTGATACCTTTCGACGGAGGCTGGATC*CCATGGCTGATATCGGATCCGAAT*
TCGAGCTCCGTCGACAAGCTTGCGGCCGCACTC GAGCACCACCACCACCACCA

29 ATGAACCGCAAAATTTTCGTTACAGGTGCTACTTCTGGTATCGGCCTTGAGTGTGCCC
GTGCTTTCGCTCAAGATGGAGATAATGTATTGATTGCTGGTCGTCGTGCAGACCGCTT
GGCGGCGATTAAGGAAGATTTTGAGCAACAATATGGCATTCGTGTGGATACGCTTGT
ACTTGATGTGAGCAAACGTGAAGATGTAGATGCTAAGGTAAAACCAGCTATCGAAGC
TTTTGGTGGCATTGATGTACTTGTAAATAATGCAGGTCTGGCACAGGGACTCGATCCA
TTTCAAGATAGTACCGTAGAAGATGCAGTCACAATGATTAATACGAATGTATTAGGC
CTTCTTTATGTGACTAAGGCTGTATTGCCGTTCATGATGGCTCAAAATAGTGGTCATA
TTGTGAACATGGGCTCTACTGCTGGAATTTATGCGTACCCAGGCGGCGCTGTGTACTG
TGCTACAAAGGCAGCTGTCAAAATGTTGGCCGATGGCATTCGTATGGATACCATAGC
TACAGATATTAAGGTTACTACCATTCAACCAGGTATCGTAGAAACTCCATTTAGTGA
AGTTCGCTTTCACGGAGATGCTGAAAGAGCAGCTTCCGTTTATGCTGGCATTGAGGCT
GTTCAACCAGAAGACGTAGCCGATGTAGTACTATATGTAACAAACCAACCTAAACGC
TTGCAAATCTCTGATGTAACCATCATGGCGAACCAACAAGCGGCAGGTTTTATG*CCAT*
GGCTGATATCGGATCCGAATTCGAGCTCCTC
GACAAGCTTGCGGCCGCACTCGAGCACCACCACCACCACCA

30 ATGAGCTATAATCTCTTAGCAGGAAAGAAGGGGATCATCTTCGGTGCTCTCAACGAA
CAGTCCATCGCTTGGCGCGTCGCAGAACGTGCTGTAGAAGAAGGCGCAGAAATCATC
CTCACCAACACGGCTGTAGCTGTTCGTATGGGCCAGCTGAATGAGCTGGGGCAGAAG
CTCAATGCTAAGGTCGTCCCCGCTGACGCTACGAAGGAAGAAGAACTCGAAGTAGTC
TTCCAGGAAGCTATGAAGAGCTTCGGCAAGGTTGACTTCGTCCTCCACTCCATCGGTA
TGAGCCCCAACGTCCGTAAGGGTCGTGCTTACGATGACCTCGACTACAAGATGCTCC
AGACGACCTTCGATATCTCGGCTGTATCTTTCCACAAGATGCTGCAGGTAGCTAAGA
AGCTCGACGCTATCGCAGAAGGTGGCTCTGTCGTCGCTCTTACTTATATCGCTGCACA
GCGTACCTTCGTCGGCTATAACGATATGGCTGATGCCAAGAGCCTCTTAGAGTCTATC
GCTCGTAGCTTCGGTTACATCTACGGTCGTGACAAGGGTGTCCGTATCAATACCGTCT
CTCAGTCTCCTACGGTCACGACTGCTGGTAGCGGTGTCAAGGGTATGTCTGACCTGCT
CGACTTCGCAGAAGACCTCTCTCCTCTGGGTAACGCCGACGCTAACGACTGCGCAGA
CTACTGTATCACGCTCTTCAGCGACCTGACGCGCAAGATCACGATGCAGAATCTCTTC
AACGACGGTGGCTTCAGCTCAATGGGTATGAGCGCTGCTGCTATCGAAGCCTTCGCT
ACGGGTCGCGCAAACCGCGAGAAG*CCATGGCTGATATCGGATCCGAATTCGAGCTCCGT*
CGACAAGCTTGCGGCCGCACTCGAGCACCACCACCACCACCA

31 ATGACTAAGCATTTACGCCGCGCCGTCGTCACCGGTGCCTCTAGCGGTATTGGCTGG
GCCATAACTGAGCGCCTAGTGACCGAGGGCTGGCAGGTGGTAGGTATTAGCCGTACC
GGCCAGGTGCCCGAGGGAGCCTTGAGTGTAAGCGCTGACTTGGCAGAGGACGGGGT
AGGGGAGGCTATCACCCAAGCCCAAAACCTGCTCGGCGGCGTCGACGCTTATGTGGG
GGCGGCTGGAAGCACCTATGAGCAGCTGGCTGCCCGCGCCGACTTGGAGCAGGTAA
ATACCCAGCTGCGCCTGCACTACCTGTCTAACTATGAGGCTATTAGCCTGCTACTGCC
GGGCATGGTCAGGGCGCGCTGGGGGCGGATTGTGCTGCTTTCCTCAGTAGTGGCTCA
AAGCGGTATGGCAGGTTTAAGCGCCTATGGGGCGGCAAAAGGTGCTCTAGAGGCGCT
GGTTAAATCGCTGGCGCTGGAGGTGGGCCGGCGCGCTATCACGGTTAATGCGGTGGC
CCCCGGTTATATCCAAACCCCCATGACCCAGTCGCTAAGCCCGCGCCAGCAAGAACG
CTACCTGCAACGCACCGGCGCCGCCAGGCCGGGCACGCCCCAGGATGTGGCCGGAC
CGGTAGCTTTTTTGCTTAGCGACGACGCCGCCTACGTTAACGGGCAGATCCTGCATGT
AGATGGGGCTATGGGGGTAGGAAAC*CCATGGCTGATATCGGATCCGAATTCGAGCTCC*
GTCGACAAGCTTGCGGCCGCACTCGAGCACCACCACCACCACCA

32 ATGAACATCTTTATTACGGGCGGAACATCGGGTATCGGCTTGGCCTTGGCGCGGTTTT
ATGCTGCCAAAGGCCATCGGGTGGGGGTGTGCGGACGAAATACGGCACGAATAGAC
AAGAGTGATGAGGTTAACAAACTGTTGCTTGCATACCAGCTGGATGTGTGTGATAAG
GACGCATTAACCGTTGCCGTAGAAGTGTTTTGTGCCGATAAGGGGTTGGACATGATG
ATTGTTGCTGCTGGATATTACCGTAACGGCGTTACTGAAGAAGTGGATTTTGAGCAG
ACTTCACAAATGTTGAAGGTTAATATCGCGGGTGCATTGAATGCGATGGAAGTGGCA
CGAGAAGCAATGAATGCATCCGGCGGACATTTGGTAGTGATTGCTTCGGTGGCAGGA
TTGTTGCATTATCCGTGCGCAAGCGTATATGCCAAGTGCAAAAGAGCATTGATACAG
ATAGCCGATGCTTATCGCCGAAGTCTTGCCGACTATCAAATAACGGTTACAACACTT

GTTCCGGGCTACATAGACACGCCACGATTGAGAGAGATATACCGTAATGACCTTTCC
AAATGTCCGTTCTGCATGCCCTTAAATCGGGCTGTTGAAACAATGACAAAGGCCATA
GCGCAGCGCAAAGAACAGGTGGTGTTTCCGCCAAAAATGCGCCTTTCCATCGCTATT
CTTTCGCTTTTACCCACTTGTTTGTTATCGGCTTTTATGCACCGAAAGACATTATGGAG
CATT*CCATGGCTGATATCGGATCCGAATTCGAGCTCC*
GTCGACAAGCTTGCGGCCGCACTCGAGCACCACCACCACCACCA

33 ATGAAGTATGCTTTAATAACTGGTGCGTCACGTGGAATAGGACGCTCTGTAGCCTTG
CTTTTAGCAGAACGGTACTCTATTATTATCAATTATCAGTCTAATGCAGAAGCTGCTC
AAGCAGTAAAACAGGAAATAGAGACCAAAGGCGGACATGTGGAACTTCTTCCTTTCG
ATGTTTCTGATCCGAAGGCGATAGAGGCAGCCATTGACACATGGGAAGCATCTCATC
CTGACGAGTTTATTTCTGTATTGGTAAACAATGCTGGTATTCGTCGTGATAATGTTAT
GTTTATGATGTCAGACGAAGACTGGCATAGCGTTCTTGACACGAATATGAATGGATT
TTTCTATATCACCCGCAGGCTTCTCAAGCACATGATGCCACGTAAGCGTGGTGGACG
AATCATCAATATGGCCTCATTGTCAGGTTTGAAAGGTTTGCCAGGGCAAGTAAATTA
TAGTGCAGCAAAAGCTGCTCTGATAGGTGCCACGAAAGCTTTGGCACAGGAAGTGGC
TGCTCGTAAGATTACGGTGAATGCTGTTGCACCTGGTTTTATTCAGACCGATATGACT
AAGGAACTGCCTGAGGACGAATTGAAGAAACTTGTCCCCGTCGGTCGTTTTGGAACA
CCAGAAGAAGTTGCAGATGTCGTGGCTTTCCTTGCCTCTGATGCTGCAGCATACATCA
CAGGTGAGGTTATCAATGTGAATGGAGGTTTCTAT*CCATGGCTGATATCGGATCCGAAT*
TCGAGCTCCGTCGACAAGCTTGCGGCCGCACTC GAGCACCACCACCACCACCA

34 ATGAGAAAAACAGCATTAATTACTGGTGCTACCAGTGGAATTGGCGAGGCTTGTGCA
CGTAAATTCGCACAAGGTGGCTACGATGTTATCATTACTGGGCGTAACAAACAGCGT
CTTGCAGCACTCAAAGTGGAGCTTGAGACAGGCGAAACAAAGGTCCTTGCACTTGCT
TTTGACGTCCGAAACCGTGCCGCAGCCACAAAGGCTATTAAGAGTTTACCGGCAGAA
TGGGCAAAGATAGATGTCCTTATCAACAATGCAGGGCTTGCTCTTGGATTGGAACCA
GAGTACGAAGGCGACTTTGAAGACTGGGATACGATGATCGATACCAACATAAAAGG
ACTCCTGACGATGACCCGTCTCATTGTTCCTAAGATGGTTGAGCGTAACAGTGGACA
CATTATTAATATAGGATCAGTGGCTGGTGACGCTGCATATGCTGGTGGAAACGTCTA
TTGTGCCACGAAGGCTGCAGTGAAAACCATCACAGATGGGCTCCGAATTGATGTTGC
TCACACTGCCGTAAGGGTTACGAATGTGAAGCCAGGGCTTGTAGAAACACACTTCTC
TAACGTCCGTTTCCACGGAGATGACAAGCGTGCGAACAGCGTTTATCATGGTATTAA
GCCGTTAACGGGTACCGATATTGCCGATGTTGCCTACTATGCAGCATCCGCTCCAGCC
CATGTGCAGATTGCAGAAGTGCTTGTTCTGGCTACACATCAGGGCAGTGGCAGCGTC
ATTCAC*CCATGGCTGATATCGGATCCGAATTCGAGCTCC*
GTCGACAAGCTTGCGGCCGCACTCGAGCACCACCACCACCACCA

35 ATGGGTTTCTTAACTGGTAAACGTATTTTAGTGGCAGGTCTTGCAAGCAACCGTTCTA
TTGCTTACGGGATCGCAAAAGCAATGAAAGAACAAGGCGCTGAACTTGCTTTCACTT
ATTTAAACGATAAATTACAACCACGCGTAGAAGAATTTGCAAAAGAATTTGGTTCTG
ACATCGTACTTCCTTTAGACGTAGCGACCGATGAAAGCATCCAAAACTGCTTTGCTG
AATTAAGCAAACGTTGGGAAAAAATTTGATGGTTTCGTACACGCTATCGCATTCGCAC
CAGGCGACCAATTAGATGGTGATTACGTAAACGCAGCAACTCGTGAAGGCTACCGTA
TCGCTCACGATATCAGTGCATTCAGCTTCGTTGCTATGGCACAAGCAGCACGTCCTTA
CTTAAATCCAAATGCAGCATTATTAACCCTTTCTTACTTAGGTGCAGAGCGCGCGATT
CCTAACTACAACGTCATGTGTTTAGCGAAAGCGTCTCTTGAAGCGGCAACTCGCGTG
ATGGCGGCTGATTTAGGTAAAGAAGGTATTCGTGTGAATGCGATCTCTGCTGGTCCT
ATCCGTACCTTAGCAGCATCAGGTATTAAAAACTTCAAGAAAATGTTTTCTGCATTTG
AGAAAACCGCAGCGTTACGCCGCACAGTTACTATCGAAGATGTGGGTAACTCAGCAG
CATTCTTATGCTCTGATTTAGCATCTGGTATTACCGGTGAAATCGTTCACGTAGATGC
AGGTTTCAGCATCACCGCAATGGGCGAATTagGCGAA*CCATGGCTGATATCGGATCCGA*
ATTCGAGCTCCGTCGACAAGCTTGCGGCCGCACTCGAGCACCACCACCACCACCA

36 ATGGCAAAAGTAATGGTAACAGGTGCTAACAAAGGCATTGGCTATGGTATCTGTAAG
TTTTTAGGCAAGAGTGGCTGGCAGGTAATCGTTGGTGCGCGTAATAGTGAGCGTGCC
GAGGAAGCGATGAAGTCATTGAAAGCTGAGGGAGTGGACGTGATTGGCTGGCAATA

CGTCAATCTATCAGACAATGCTTCTTTGGAGCAAACAGCAAAAGAAGTAAAAGAGA
AATATCATGACTTGGAACTATTGGTAAACAATGCTGGTATTCCTGGCGATATGAAAG
TAGCAAGTTATGAGTCAGAGCTAAAGGATGTAATAGATACCGTACAAGTAAACTATG
TCGGAACATTCTGCTTGACAAAGGCACTTACCCCATTACTCTCTGCAAACAAAGGAA
GAATTGTGAACATAACCGTACCATCTGAAGTAAGTCCTTATTGGCATCCAATGGCTTA
CGTAGCCAGCAAAGCAGCACAGAATGCAATGACCAGCATTATGGCTATGGAATTTGA
GAAGAACAACATACCTGTTGAAATCTTCAATATCCATCCTGGTGCTACGACAACCGA
TTTAAATAACCATTATACAGGACCAGGTTCTCACTCGATAGATGTTGTTAGTGAGAA
GATTGCAGAAGTTATTAATGATGGGAAAAAGCATCAAGGAGAGTTTGTTGAACTATA
TCCTATCGTAGACGAAGGACGACCATGGCTGATATCGGATCCGAATTCGAGCTCCGT
CGACAAGCTTGCGGCCGCACTCGAGCACCACCACCACCACCAC

37 ATGCAAAGAACAGCGTTAGTAACTGGCGCAACTGCCGGATTTGGTGCGGCAATTTGT
CGCACACTTATTGAAAATGGTTATCGTGTAATTGGCACGGGTCGCCGTGTGGCTCGTT
TAGAACAATTACAGCAAGAATTAGGTGAAAACTTCCACTTTCTTGCCTTTGATATTTC
AGATCGCCAAGCAACAGAAGATGCTTTCCATTCCCTTCCCACTAATTGGCAATCCATT
GATTTATTGGTGAATAATGCAGGTTTAGGATTAGGCTTAGAAAGTGCCGATAAAGCG
AGTTTAGACGATTGGATGCAAATGATTGATACCAATATTAAAGGACTCGTCACCATC
ACTCGTCTTGTATTACCACAAATGGTTGAACGCAATTCAGGTCATATTATTAATTTAG
GCTCAATTGCAGGTACTTATCCTTATCCAGGTGGCAATGTATACGGTGGCACTAAAG
CTTTTATTAAACAATTTAGTTTAAATCTTCGAGCCGATCTTGCTGGAACTCAAATTCG
CGTGACCAATGTAGAGCCTGGTCTTTGTGGTGGAACTGAATTTTCTAATATCCGCTTT
AAAGGTGATGATGCCCGAGCAAAAAAACTCTATGAAAATGTGGAATATGTCAGTCCA
CAAGATATTGCTAATATTGTGTTATGGCTCAATCAACAACCTGAACATGTCAATATTA
ATCGCATTGAAGTGATGCCAACTGCCCAAACCTTTGCACCACTTAATGTCGCAAGA*C*
CATGGCTGATATCGGATCCGAATTCGAGCTCCGTCGACAAGCTTGCGGCCGCACTCGAGC
ACCACCACCACCACCAC

38 ATGCACTTAGAGGGTAAAGTAGCTCTCGTAACTGGCGCCTCTCGTGGTATCGGTCG
CGCCGTTGCTATTCAACTTGCACAATCTGGTGCTGATGTTGCTGTTAACTATAGTG
GCAGTGAAGCTGCTGCTCAAGAAACAGTGGACGCTATCTTAGCATTGGGGCGCAA
AGCGATTAAAATTAAAGCTAATGTTGCAAATGCTGAAGAAGTTGCAGCAATGGTT
GAAGAAACGCATAAAACATTTGGTCACATCGATATCTTGGTCAATAATGCAGGTA
TCACACGTGATGGTTTACTTATGCGTATGAAAGATGAAGACTTTGATGCCGTTATC
GATATCAACCTTAAAGGTGTATATTTAGTAACAAAAGCAGTATCCAAAATCATGAT
GAAACAACGTGCTGGTCATATTATTAACATGACTTCTGTTGTTGGTTTGATGGGTA
ATGCTGGTCAAGCTAACTATGCAGCTTCTAAAGCTGGTGTAATCGGTTTTACTAAA
TCTTGTGCAAAAGAGTTAGCTAGCCGTGGTATCACAGTTAATGCAATCGCACCTGG
TTTTATCAATACTGATATGACAGATGTATTGCCTGAAAAGGTAAAAGAAGCTATGG
TAACACAAATTCCGTTGGGCCGTATGGCTAAAGCTGAAGAAGTGGCTGCCGTAAC
AACATTCCTTGCTAGTGATTTTGCTAGCTATATTACAGGTCAAGTCATCAATGTAG
ATGGCGGCATG*CCATGGCTGATATCGGATCCGAATTCGAGCTCCGTCGACAAGCTTGC*
GGCCGCACTCGAGCACCACCACCACCACCAC

Table S3. Amino acid sequences of the 37 retrieved SDRs (excluding the 20 amino acids and hexa histidine tag coded for by the pET29a vector).

SDR #	Amino acid sequence
1	MNLLANKVAIITGAGRGIGRAIALKYAQEGASVVITDLKIDETVEAFVKELEGLGVKA<br>KAYASNAANFEDAHKLVEAVVADFGRIDVLVNNAGITRDGLMMRMTEEQWDLVINV<br>NLKSAFNLIHAVTPVMVKQRSGSIINMASVVGVSGNAGQANYSASKAGMIGLAKSIA<br>KELGARGIRANAIAPGFIITDMTGALSEEVRKQWEVQIPLRRGGTPEDVANVATFLASD<br>LSSYVSGQTIHVCGGMNM
2	MSTQDLSGKIALVTGASRGIGAAIADTLAVAGAKVIGTATSESGAAAISERLAQWGGE<br>GRALNSAEPETIENLIADIEKEFGKLDILVNNAGITRDNLLMRMKEEEWDDIMQVNLK<br>SVFRASKAVLRGMMKQRAGRIINITSVVGVMGNAGQTNYAAAKAGLIGFSKSMARE<br>VGSRGITVNCVAPGFIDTDMTRALPEETRKTFEAQTSLGKFGEAQDIADAVLFLASDQ<br>AKYITGQTLHVNGGMLMPPWLISXPEFQHT
3	MAHNIFVTGATSGIGLCIAEAYAKHGDNVLISGRRAELLGEVQARLSKEYGVRVETLV<br>LDVRSREDVESKVPAAIEAFGGVDVLVNNAGLAQGLDPFQDSAVDDAVTMIDTNVK<br>GLLYVTKAVLPFMIDKNEGHIVNMGSTAGIYAYPNGAVYCATKAAVKTLSDGIRMDT<br>ITTDIKVTTIQPGIVETPFSEVRFHGDAERAKSVYAGIDAIQPEDVADVVLYVTNQPKRL<br>QISDVTIMANQQAAGFMV
4	MSKVAIVTGAGQGIGFAIAKRLVQDGFKVGVLDYNAETAEKAVAELSADKAFAVVA<br>DVSKQAEVAAAFQKVVDHFGDLNVVVNNAGVAPTTPLDTITEEQFNRTFAINVGGVI<br>WGAQAAQAQFKALGHGGKIINATSQAGVVGNPNLTVYGGTKFAVRGITQTLARDLA<br>DSGITVNAYAPGIVKTPMMYDIAHEVGKNAGKDDEWGMQTFAKDITLKRLSEPEDVA<br>AAVSF LAGPDSNYITGQTIIVDGGMQFH
5	MSETILVTGASAGFGQAICRRLVADGYRVIGSARRIDKLQALQEELGEAFYPLQMDVT<br>DLSQVDHALASLLKAWEKVDVLVNNAGLALGLAPAYEAEVADWLTMIQTNIVGLTY<br>LTRKILPQMVERNDGYIINLGSTAGTVPYPGANVYGASKAFVKQFSLNLRADLAGKKI<br>RVSNIEPGLCEGTEFSSVRFKGDEKRVEALYRDAHAIQSEDIANTVAWLIQQP<br>KHVNVNRIEIMPVSQTFGPQPVYR
6	MYSELKGKVAVITGGSKGIGTAIAKRFGQEGMKVVINYNSDAAGAELAAEAVRCAG<br>GEAVTVKAHVGTEEGVQSLVDAAVENYSGIDVWINNAGMENKVATHEMPLSDWER<br>VINVNLTGVFLGTRAALTYFMEHDVKGSIVNMSSVHEQIPWPTFAHYCASKGGIKLFT<br>QTVAMEYAKYGIRVNAVGPGAINTPINAKKFSDPVQYEETMSMVPMKRIGKPEEVAA<br>CVAWLASDEASYVTGITLFVDGGMTLYPAFQDGKG
7	MPHKNDVQVALISGGTSGIGFATAKLLLQEGWCVVINGRDEQAGQRAKMKLRRYSS<br>KVRYVKGDVSSVSDCQRIVKETVDFFGSISALVTAAGYYEEELLADVSESAFDEMFGT<br>NVKGTVFLCQAALPYLRQVKGSIVTVSSDAGLQGNVACSVYGASKGAIVSFTKSLSLE<br>MAPHEVRVNCVCPGDVDTSLVDKQIAQSHQDAEQAKEEMGQHYPLGRI<br>AKPHEIGEVIAFLISSKASFVTGAAWTIDGGLTS
8	MTKRVLVTGVSSGIGLAQARLFLENGYQVYGVDQGEKPDLQGNFHFLQRDLTLDLEP<br>IFDWCPQVDILCNTAGILDDYKSLLEQSAQEIQEIFEINYVTPVELTRYYLTQMLEYKR<br>GTIINMCSIASSLAGGGGHAYTSSKHALAGFTKQLALDYAEAGIQVFGIAPGAVKTGM<br>TAADFEPGGLADWVASETPIKRWIEPEEVAEVSLFLASGKASAMQGQILTIDGGWSL
9	MSWVIVTGANGGIGEATVHQLIKNGYSVFAADLAEQPIASFGTYGDAIFRYRAVDVTS<br>EESVTALAEAVAALDEPITGAVLAAGIAHSQPLLETSFATWKRLHAVNSDGVFLCLRE<br>FARIMIDQQESDPTNSRSLVTVASNAARVPRAEFGAYGASKASAARVSSSFGLQLAAH<br>GIRVNSVCPGTTRTPMVTDAWEGEDRSALPVAGNPQTFRLGIPLGRIADPADIAAVNA<br>FL ISEAARHITMQEIVA
10	MTENTHIPASPFLPHTGDGKVAVVTGASSGIGRATVQQLVASGWTVYALARRTDRLY<br>TLYAETGAHPVTCDVTDEQSVQFVAEQILEEQGTIDALVNIAGGAIGVDKVAEGKPDD

YLKMYQMNVLGILHMVRAFAEALRQNGYGTILNLTSTAAEHGYEGGAGYNAAKFGA
RGLTEALRLEEAENNIRVIEICPGMVHTEEFSLNRLGSKEAAERVYAGVEKPLTAEDVA
QTVTFALNVPHHVNLDRITIRPVAQPSQFKVIRKEGP

11 MPKSLQRPVVLLTGASSGIGYDVAPLLVRYGYIVYGAARRVEKIEELASEGVKALSLD
VTDEASMEAAVQQIIDAEGRIDVLINNAGYGSYGAIEDVPIDEARRQFEVNLFGLARLT
QLVLPHMRARGSGRILNISSMAGRITSPLGAWYHATKYALEAFSDALRMEVEEFGIDV
VIIEPGGIKTPWGLIAADHLEESSRNGVYAAQAQRVAANMRRLYSPSSNLSEPKVISNA
ILRALEARRPKTRYLVGFGAKPSVFLHTVLPDRLFDKVARRIF

12 MSNAPEVRGLFLKALGRPVIVAPSSAEPTVTFDGPLTEVCSCSLKETELPVVVRAGEET
FEVRATSTGERTINGRVALVTGGAQGFGAEIARGLVDAGCFVFIADLNGEGAAAKAA
ELGGEGVAHPITVNVADEESVAAMAAEIERVTGGLDLVVSNAGIVRAGSVLEQDASA
FRLSTDINYVAFFLVTKHLGQLLARQHSTAPEWLTDIIQINSKSGLVGSNKNAAYAGSK
FGGIGLVQSFALEMVAHGVKVNAICPGNFYDGPLWSDPDRGLFVQYLNSGKVPGAKT
VADVKEFYEAKVPMRRGAQGIDVLRAIFYIVEQEYETGQAVPVTGGQVMLS

13 MNTSRRVVVTGASTGIGQATARLLAKRGWKVVAVARRRERLEALAEQIGCEYWAAD
LTDEAQVKEMAAHVLEGGPVDAVVNNAGGAIGVDRVAEGDPARWSAMFERNVLTA
LHCSRAFLPGMRERGGDLVFLTSTAAHDTYPGGGGYVAAKHAERIIANTLRQELVGE
PVRVIEIAPGMVRTEEFSLNRLGSQEAADRVYEGVAAPLVAEDIAEAIVWTLERPSHV
NIDSMIVRPVAQATNTLVARKTA

15 MDIADAEGVKKTVAQILEECGDVNVLINCAGRISSVPFTEVDDKEWNNTINTNLTGTY
NVTHALWQHFIDRGGARIVNVSSVAGKIGGGLLGTVAYASSKAGMNGFTKAIAKEGG
KYGISCNAVCPSFTITDMTTALSNDEEKYKKVVGIIPLGRPAQASEPAQMVLFFASDAA
SFVNGEVGDCDGGIVME

16 MSENTPKTVLVTGGNRGIGYEIAKEFQAAGHNVCITYRSGEAPEEFFAVKADVRDADS
INEAFKEIEAEFGPVEVLVANAGITRDMLLMRMKESDFTDVVDTNLTGSFRVVQRAIK
GMLKLKRGRIILVSSVVGLYGSPGQVNYSASKAALVGMARSITRELGGRNITANVVAP
GFINTAMTEVLPEETKKNYLASIPAGRFAXAEEVARVIRWLASDEASYISG
AVIPVDGGLGMGH

17 MELKNKNVFVTGSTRGIGLAVAHKFASLGANVVLNGRSEISEDLLAQFADYGVTVVG
ISGDISNGEDAQRMVAEAIEKLGSVDVLVNNAGITNDKLMLKMTEEDFERVLKINLTG
AFNMTQAVLKPMSKARQGAIINMSSVVGLMGNIGQANYAASKAGLIGFTKSVAREVA
ARGVRVNAIAPGFIESDMTDAIPEKMKDAMLAQVPMKRIGQAEEVAEVAAFLAGQEY
LTGQT IAIDGGMTMQ

18 MAILITGASAGFGAAMCRTFVAAGYHVIGAARREDKLQQLAEELGEQFYPLEMDVSR
TESIQNALNSLPEHLSEIDCLINNAGLALGLDTADKADFGDWETMIQTNIIGLTFLTRQI
LPQMVARKQGYIINLGSIAGSYAYSGSNVYGATKAFVRQFSMNLRAELADKNIRITNIE
PGLCGDTEFSNVRFKGDDQRAAEVYENVEFIQPQDIADTALWLYQRPARMNVNSIEIM
PVAQTFAGMKVYRDEPAPAKEETFEKQSMSLFGKIKSWFK

19 MNDWLNIKGKTVLVTGASSGIGKAIVEELLELGVNVANFDLSDNDLRHPNLLFVKVD
VTSRSEVEEGVAKIVERFGNIDAVVNNAGINIPRLLIDAENPKGPYELDDETFEKVTMI
NQKGLYLVSQAVGRILVKNGKGVIVNMASEAGLEGSEGQSAYAATKAAVYSYTRSW
AKELGKHGVRVVGIAPGIMEATGLRTLSYEEALAYTRGKTVEDIRAGYASTSTTPLGR
SGKLREVADLVAFYISDRSSYITGVTTNIAGXKTRG

20 MYLCKQIYHTDKHRKTNMAKVLVTGANKGIGYGICKFLGKSGWQIIVGARNSERAEE
AMKSLKAEGVDVIGWQYVNLSDNASLEQTAKEVKEKYHDLELLVNNAGIPGDMEVA
SYESELKDVIDTVQVNYVGTFCLTKALTPLLSANKGRIVNITVPSEVSPYWHPMAYVA
SKAAQNAMTSIMAMEFEKNNIPVEIFNIHPGATTTDLNNHYTGPGSHSIDVVSE
KIAEVINDGGKHQGEFVELYPI

21 MKLLEGKTALITGAARGIGKAIALKFAEEGANIAFTDLVIDENGKATEAEIAAKGVKV
KGYASNAADFAQSEEVVKQVKEEFGSIDILVNNAGITKDGLMLRMTEQQWDAVIAVN

LKSAFNFIHACVPVMMRQRNGSIINMASVVGVHGNGGQANYAASKAGMIALAKSVA
QEMGPKGIRANAIAPGFIDTAMTQALNDDIRKEWTSKIPLRRGGTVDDIANTAV
YLGSELSSYVSGQVIQVDGGMNM

22 MMTSLRKKIIAIGGALAYPSISLTPDITREHFSGKWVLVTGASHGIGRALTEKIINAGAN
VFLIARSEADLRLLCAKAKQMGSSADYCAIDLRDREKLEQLCQKLRETLPRLDYFFCN
AGKSIHRKINDAQDRLHDYDRTMDLNYRSLVALSLAILPALKASKGSIIYSSSVSTLYP
MAPGWSAYHASKSAANTWCETANSEFAPLGVHVQIAYLPLVHTAMSDVNEQYKHLP
AYTPADAANILLKLAIRKVRTYKPWWAKLSAPIAYLFAPIIHLYYKR

23 MKKAIVVGASSGIGHEVARLLIAQGWAVGVAARRIDKLTDLQAMAPERVYTAQIDVN
NEDAETSLLQLIERMNGIDLYFHAAGIGWQNPSLNADIELKTMETNAVGFTRMIGCAY
RYFANKGGGHIACITSIAGTKGLGPAPAYSATKAMQNTYLQALEQLAACKHHNIHFT
DIRPGFVDTPLLAGTSHLPMLMTTEKVARSIIKAINSRRHICVIDSRWCVLTY
LWRHIPNWIWRRMKLCQ

24 MKMEDTIKKGIIIGATGGIGRQLAKELAQRLEHLILVSRDADKLSQVQKELTGSKAQLS
ILTLDMLDQVALEAFVESLDADLLVNCAGLAYFSRESDLDSASEQDLWQVNYHSSVQ
LIKQVVQKNQKIQLVQLSSLAALFPHPYLAAYSASKAALQTFTLALQEELRQSESQVQ
LGLYILGPVQTAIFPPKLVEALGGSRLQMKSEKVAQQLIRFIERDTSYEII
GLRYRLLVWLGRLLPQRWIIRLLAIYLKKGT

25 MSYNLLKGKRGIIFGALNEQSIAWKVAERAVEEGASITLSNTPIAVRMGTVNSLAEKL
NCEVIAADATSVEDLENVFKRSMEILGGKIDFVLHSIGMSPNVRKHRTYDDLDYNML
NTTLDISAVSFHKMIQSAKKLDAISDYGSILALSYIAAQRTFFGYNDMADAKALLESIA
RSFGYIYGREKNVRINTISQSPTMTTAGQGVKGMDKLYDFANRMAPLGNASAAECAD
YCIVMFSDLTKKVTMQNLYHDGGFSNIGMSLRAMTTYEKGIGDEYKDENGKIIYG

26 MSETILITGSNRGIGKAVALGLAQDGFDIVVHCRSRRDEAEAVAEEIRALGRNARVLQ
FDVSDREACREILTADIEANGTYYGVVLNAGLTRDNAFPAFTDDDWDLVLRTNLDGF
YNVLHPLTMPMIRRRKAGRIVCMASVSGLTGNRGQVNYSASKAGLIGAAKALAVELA
KRKITVNCVAPGLIDTDIIDENVPVEEILKAVPAARMGLPEEVAHAVRFLMDE
KAAYITRQVIAVNGGLC

27 MAYNLLKGKRGVIFGALNDMSIAWKVAERCAEEGATLVLSNTEMALRMGSLDELSK
KINAPVIAADATNYDDLENVFVKAQELLGGKIDFVLHSIGMSPNVRKHRTYDDLDYN
WLNKTLDISAISFHKMLQAAKKVDAIAEYGSVVALTYVASHRTFFGYNDMADAKSLL
ESIARSFGYIYGREKNVRINTISQSPTETTAGKGIKDIDNMMDFADKMSPLGNATADEC
ADYCVTLFSDLTRKVTMQTLFHDGGFSNMGMSLRAMNQYSKTLD

28 MNIAIITGASRGIGKAAAKRFAREGYSLLLNCEKNWTLLEELKKEIQSDLPENCPEIFLC
KDLGTKKGLSRILEGKSLSKLILIANSGKDAIKLLQDCREEETKALLETNLLQPFILCQK
LLPYLLQAEEGRILFSSSVWGNVGASMESLYSLTKGGISTFAKALGKELAPSHISVNAV
AFGAVDTDMNSWLSTEEKQSLEEGIPYGRMATVEEAADFLYLLSQAPLYLTAQVI
PFDGGWI

29 MNRKIFVTGATSGIGLECARAFAQDGDNVLIAGRRADRLAAIKEDFEQQYGIRVDTLV
LDVSKREDVDAKVKPAIEAFGGIDVLVNNAGLAQGLDPFQDSTVEDAVTMINTNVLG
LLYVTKAVLPFMMAQNSGHIVNMGSTAGIYAYPGGAVYCATKAAVKMLADGIRMD
TIATDIKVTTIQPGIVETPFSEVRFHGDAERAASVYAGIEAVQPEDVADVVLYVTNQPK
RLQIS DVTIMANQQAAGFM

30 MSYNLLAGKKGIIFGALNEQSIAWRVAERAVEEGAEIILTNTAVAVRMGQLNELGQKL
NAKVVPADATKEEELEVVFQEAMKSFGKVDFVLHSIGMSPNVRKGRAYDDLDYKML
QTTFDISAVSFHKMLQVAKKLDAIAEGGSVVALTYIAAQRTFVGYNDMADAKSLLESI
ARSFGYIYGRDKGVRINTVSQSPTVTTAGSGVKGMSDLLDFAEDLSPLGNADANDCA
DYCITLFSDLTRKITMQNLFNDGGFSSMGMSAAAIEAFATGRANREK

31 MTKHLRRAVVTGASSGIGWAITERLVTEGWQVVGISRTGQVPEGALSVSADLAEDGV
GEAITQAQNLLGGVDAYVGAAGSTYEQLAARADLEQVNTQLRLHYLSNYEAISLLLP

	GMVRARWGRIVLLSSVVAQSGMAGLSAYGAAKGALEALVKSLALEVGRRAITVNAV APGYIQTPMTQSLSPRQQERYLQRTGAARPGTPQDVAGPVAFLLSDDAAYVNGQILH VDGAMGVGNP
32	MNIFITGGTSGIGLALARFYAAKGHRVGVCGRNTARIDKSDEVNKLLLAYQLDVCDK DALTVAVEVFCADKGLDMMIVAAGYYRNGVTEEVDFEQTSQMLKVNIAGALNAME VAREAMNASGGHLVVIASVAGLLHYPCASVYAKCKRALIQIADAYRRSLADYQITVT TLVPGYIDTPRLREIYRNDLSKCPFCMPLNRAVETMTKAIAQRKEQVVFPPKMRLS IAILSLLPTCLLSAFMHRKTLWSI
33	MKYALITGASRGIGRSVALLLAERYSIIINYQSNAEAAQAVKQEIETKGGHVELLPFDV SDPKAIEAAIDTWEASHPDEFISVLVNNAGIRRDNVMFMMSDEDWHSVLDTNMNGFF YITRRLLKHMMPRKRGGRIINMASLSGLKGLPGQVNYSAAKAALIGATKALAQEVAA RKITVNAVAPGFIQTDMTKELPEDELKKLVPVGRFGTPEEVADVVAFLASDAAAYITG EVI NVNGGFY
34	MRKTALITGATSGIGEACARKFAQGGYDVIITGRNKQRLAALKVELETGETKVLALAF DVRNRAAATKAIKSLPAEWAKIDVLINNAGLALGLEPEYEGDFEDWDTMIDTNIKGLL TMTRLIVPKMVERNSGHIINIGSVAGDAAYAGGNVYCATKAAVKTITDGLRIDVAHTA VRVTNVKPGLVETHFSNVRFHGDDKRANSVYHGIKPLTGTDIADVAYYAASAPAHVQ IAE VLVLATHQGSGSVIH
35	MGFLTGKRILVAGLASNRSIAYGIAKAMKEQGAELAFTYLNDKLQPRVEEFAKEFGSD IVLPLDVATDESIQNCFAELSKRWEKFDGFVHAIAFAPGDQLDGDYVNAATREGYRIA HDISAFSFVAMAQAARPYLNPNAALLTLSYLGAERAIPNYNVMCLAKASLEAATRVM AADLGKEGIRVNAISAGPIRTLAASGIKNFKKMFSAFEKTAALRRTVTIEDVGNSAAFL CSDLASGITGEIVHVDAGFSITAMGELGE
36	MAKVMVTGANKGIGYGICKFLGKSGWQVIVGARNSERAEEAMKSLKAEGVDVIGW QYVNLSDNASLEQTAKEVKEKYHDLELLVNNAGIPGDMKVASYESELKDVIDTVQVN YVGTFCLTKALTPLLSANKGRIVNITVPSEVSPYWHPMAYVASKAAQNAMTSIMAME FEKNNIPVEIFNIHPGATTTDLNNHYTGPGSHSIDVVSEKIAEVINDGKKHQGEFVELYP IVDEGR
37	MQRTALVTGATAGFGAAICRTLIENGYRVIGTGRRVARLEQLQQELGENFHFLAFDIS DRQATEDAFHSLPTNWQSIDLLVNNAGLGLGLESADKASLDDWMQMIDTNIKGLVTI TRLVLPQMVERNSGHIINLGSIAGTYPYPGGNVYGGTKAFIKQFSLNLRADLAGTQIRV TNVEPGLCGGTEFSNIRFKGDDARAKKLYENVEYVSPQDIANIVLWLNQQP EHVNINRIEVMPTAQTFAPLNVAR
38	MHLEGKVALVTGASRGIGRAVAIQLAQSGADVAVNYSGSEAAAQETVDAILALGR KAIKIVSKIMMKQRAGHIINMTSVVGLMGNAGQANYAASKAGVIGFTKSCAKELAS RGITVNAIAPGFINTDMTDVLPEKVKEAMVTQIPLGRMAKAEEVAAVTTFLASDFA SYITGQVINVDGGMKANVANAEEVAAMVEETHKTFGHIDILVNNAGITRDGLLMR MKDEDFDAVIDINLKGVYLVTKA

Table S4. Table of primers used to retrieve SDRs from the oral metagenome. Italicised sequences match MCS from pet29a to facilitate Gibson assembly.

SDR #	Primers
1 FWD	*TAAGAAGGAGATATACAT*ATGAATCTACTTGCTAACAAGGTAGCC
1 RVR	*ATCCGATATCAGCCATGG*CATATTCATCCCACCGCAGACG
2 FWD	*TAAGAAGGAGATATACAT*ATGAGTACACAAGATTTGAGCGGCAA
2 RVR	*ATCCGATATCAGCCATGG*AGGCATCAACATGCCGCCGT
3 FWD	*TAAGAAGGAGATATACAT*ATGGCTCATAATATTTTTGTCACTGGTGC
3 RVR	*ATCCGATATCAGCCATGG*CACCATAAAGCCTGCCGCTTGT
4 FWD	*TAAGAAGGAGATATACAT*ATGTCTAAAGTAGCTATTGTTACAGGTGC
4 RVR	*ATCCGATATCAGCCATGG*ATGGAATTGCATACCACCATCGAC
5 FWD	*TAAGAAGGAGATATACAT*ATGTCAGAAACGATTTTAGTAACAGGAGC
5 RVR	*ATCCGATATCAGCCATGG*ACGATAAACGGGTTGAGGACCAAAG
6 FWD	*TAAGAAGGAGATATACAT*ATGTACAGTGAATTAAAAGGAAAGGTAGCC
6 RVR	*ATCCGATATCAGCCATGG*ACCCTTACCGTCTTGAAAAGCCG
7 FWD	*TAAGAAGGAGATATACAT*ATGCCTCATAAAAATGATGTTCAAGTGGCA
7 RVR	*ATCCGATATCAGCCATGG*ACTCGTAAGACCTCCATCAATCG
8 FWD	*TAAGAAGGAGATATACAT*ATGACTAAACGCGTCTTAGTTACAGGTG
8 RVR	*ATCCGATATCAGCCATGG*CAAAGACCAGCCACCATCAATAGTC
9 FWD	*TAAGAAGGAGATATACAT*ATGAGCTGGGTCATTGTGACCGGC
9 RVR	*ATCCGATATCAGCCATGG*GGCGACAATCTCCTGCATGGTG
10 FWD	*TAAGAAGGAGATATACAT*ATGACTGAGAACACCCACATTCCTGC
10 RVR	*ATCCGATATCAGCCATGG*GCCCTCCTTGCGAATCACCTTG
11 FWD	*TAAGAAGGAGATATACAT*ATGCCCAAGTCACTGCAACGCC
11 RVR	*ATCCGATATCAGCCATGG*GAAGATACGGCGCGCCACCTTG
12 FWD	*TAAGAAGGAGATATACAT*ATGAGTAACGCTCCCGAGGTACGTG
12 RVR	*ATCCGATATCAGCCATGG*GAGAGCATCACCTGGCCGCC
13 FWD	*TAAGAAGGAGATATACAT*ATGAACACTTCTCGTCGCGTCGTTGTTAC
13 RVR	*ATCCGATATCAGCCATGG*GGCGGTCTTCCTGGCCACGAG
14 FWD	*TAAGAAGGAGATATACAT*ATGGGAACAGCGCTGGTCACCGG
14 RVR	*ATCCGATATCAGCCATGG*CATGTGGGGGACGGAGCGCATG
15 FWD	*TAAGAAGGAGATATACAT*ATGGACATTGCAGATGCCGAGGGCGT

15 RVR	*ATCCGATATCAGCCATGG*TTCCATCACGATGCCGCCGTCGCA
16 FWD	*TAAGAAGGAGATATACAT*ATGAGCGAGAACACTCCCAAGACCGTGCT
16 RVR	*ATCCGATATCAGCCATGG*GTGACCCATGCCCAGGCCGCCAT
17 FWD	*TAAGAAGGAGATATACAT*ATGGAACTTAAAAATAAAAATGTTTTG
17 RVR	*ATCCGATATCAGCCATGG*TTGCATAGTCATTCCGCCA
18 FWD	*TAAGAAGGAGATATACAT*ATGGCAATTTTGATTACCGGG
18 RVR	*ATCCGATATCAGCCATGG*CTTAAACCAAGATTTAATTTTTCCAAAC
19 FWD	*TAAGAAGGAGATATACAT*ATGAATGATTGGTTAAACATAAAAGGC
19 RVR	*ATCCGATATCAGCCATGG*ACCGCGAGTTTTCCCTCC
20 FWD	*TAAGAAGGAGATATACAT*ATGTATCTTTGCAAGCAAATATATCACA
20 RVR	*ATCCGATATCAGCCATGG*GATAGGATATAGTTCAACAAACTCTC
21 FWD	*TAAGAAGGAGATATACAT*ATGAAATTATTGGAAGGTAAGACAGC
21 RVR	*ATCCGATATCAGCCATGG*CATATTCATACCGCCATCAACC
22 FWD	*TAAGAAGGAGATATACAT*ATGATGACGTCTCTCAGAAAAAAGAT
22 RVR	*ATCCGATATCAGCCATGG*TCGCTTATAATATAGGTGAATAATCGG
23 FWD	*TAAGAAGGAGATATACAT*ATGAAAAAAGCAATCGTCGTAGGC
23 RVR	*ATCCGATATCAGCCATGG*TTGACATAGCTTCATTCGCCTCC
24 FWD	*TAAGAAGGAGATATACAT*ATGAAGATGGAAGATACAATTAAAAAAGGG
24 RVR	*ATCCGATATCAGCCATGG*AGTCCCTTTTTTAAATATATAGCAAGGAG
25 FWD	*TAAGAAGGAGATATACAT*ATGAGTTATAACTTATTAAAAGGTAAAAGAGG
25 RVR	*ATCCGATATCAGCCATGG*TCCGTAGATAATCTTTCCGTTCTC
26 FWD	*TAAGAAGGAGATATACAT*ATGAGTGAAACTATTTTAATTACAGGGTC
26 RVR	*ATCCGATATCAGCCATGG*ACACAAACCTCCGTTCACAGC
27 FWD	*TAAGAAGGAGATATACAT*ATGGCTTATAACTTATTAAAAGGAAAACGTG
27 RVR	*ATCCGATATCAGCCATGG*ATCCAAAGTCTTACTATATTGGTTCATAG
28 FWD	*TAAGAAGGAGATATACAT*ATGAACATTGCTATTATTACCGGTGC
28 RVR	*ATCCGATATCAGCCATGG*GATCCAGCCTCCGTCGAAAGG
29 FWD	*TAAGAAGGAGATATACAT*ATGAACCGCAAAATTTTCGTTACAGG
29 RVR	*ATCCGATATCAGCCATGG*CATAAAACCTGCCGCTTGTTGG
30 FWD	*TAAGAAGGAGATATACAT*ATGAGCTATAATCTCTTAGCAGGAAAG
30 RVR	*ATCCGATATCAGCCATGG*CTTCTCGCGGTTTGCGCGA

31 FWD	*TAAGAAGGAGATATACAT*ATGACTAAGCATTTACGCCGCG
31 RVR	*ATCCGATATCAGCCATGG*GTTTCCTACCCCCATAGCCC
32 FWD	*TAAGAAGGAGATATACAT*ATGAACATCTTTATTACGGGCGGAAC
32 RVR	*ATCCGATATCAGCCATGG*AATGCTCCATAATGTCTTTCGGTGC
33 FWD	*TAAGAAGGAGATATACAT*ATGAAGTATGCTTTAATAACTGGTGCTTC
33 RVR	*ATCCGATATCAGCCATGG*ATAGAAACCTCCATTCACATTGATAACCT
34 FWD	*TAAGAAGGAGATATACAT*ATGAGAAAAACAGCATTAATTACTGGTGC
34 RVR	*ATCCGATATCAGCCATGG*GTGAATGACGCTGCCACTGC
35 FWD	*TAAGAAGGAGATATACAT*ATGGGTTTCTTAACTGGTAAACGTATTTTAG
35 RVR	*ATCCGATATCAGCCATGG*TTCGCCTAATTCGCCCATTGCG
36 FWD	*TAAGAAGGAGATATACAT*ATGGCAAAAGTAATGGTAACAGGTGC
36 RVR	*ATCCGATATCAGCCATGG*TCGTCCTTCGTCTACGATAGGATA
37 FWD	*TAAGAAGGAGATATACAT*ATGCAAAGAACAGCGTTAGTAACTGG
37 RVR	*ATCCGATATCAGCCATGG*TCTTGCGACATTAAGTGGTGCAAAGG
38 FWD	*TAAGAAGGAGATATACAT*ATGCACTTAGAGGGTAAAGTAGCTCT
38 RVR	*ATCCGATATCAGCCATGG*CATGCCGCCATCTACATTGATGAC

2. Figures

Figure S1.

A. Sequence alignments for SDRs 3,4,11,17,31,37. The catalytic triad Ser-Tyr-Lys and a fourth residue considered to be important are all marked red. The TGxxxGxG motif important in cofactor binding is marked in purple.

```
11      MPKSLQRPVVLLTGASSGIGYDVAPLLVRYGYIVYGAARRVEKIEELASE-------GVK
3       -----MAHNIFVTGATSGIGLCIAEAYAKHGDNVLISGRRAELLGEVQARLSKEYGVRVE
37      -----MQRTALVTGATAGFGAAICRTLIENGYRVIGTGRRVARLEQLQQEL----GENFH
4       -----MSKVAIVTGAGQGIGFAIAKRLVQDGFKVGVLDYNAETAEKAVAELS-AD--KAF
17      --MELKNKNVFVTGSTRGIGLAVAHKFASLGANVVLNGRS-EISEDLLAQFA-DYGVTVV
31      --MTKHLRRAVVTGASSGIGWAITERLVTEGWQVVGISRTGQVPEGALS-----------
                  .:**:  *:*  :       *  *

11      ALSLDVTDEASMEAAVQQIIDAEGRIDVLINNAGYGS-YGAIEDVPIDEARRQFEVNLFG
3       TLVLDVRSREDVESKVPAAIEAFGGVDVLVNNAGLAQGLDPFQDSAVDDAVTMIDTNVKG
37      FLAFDISDRQATEDAFHSLPTNWQSIDLLVNNAGLGLGLESADKASLDDWMQMIDTNIKG
4       AVVADVSKQAEVAAAFQKVVDHFGDLNVVVNNAGVAP-TTPLDTITEEQFNRTFAINVGG
17      GISGDISNGEDAQRMVAEAIEKLGSVDVLVNNAGITN-DKLMLKMTEEDFERVLKINLTG
31      -VSADLAE-DGVGEAITQAQNLLGGVDAYVGAAGSTY-EQLAARADLEQVNTQLRLHYLS
         :  *: .       .         ::  :. **             ::    :  :  .

11      LARLTQLVLPH-MRARGSGRILNISSMAGRITSPLGAWYHATKYALEAFSDALRMEVEEF
3       LLYVTKAVLPF-MIDKNEGHIVNMGSTAGIYAYPNGAVYCATKAAVKTLSDGIRMDTITT
37      LVTITRLVLPQ-MVERNSGHIINLGSIAGTYPYPGGNVYGGTKAFIKQFSLNLRADLAGT
4       VIWGAQAQAQAQFKALGHGGKIINATSQAGVVGNPNLTVYGGTKFAVRGITQTLARDLADS
17      AFNMTQAVLKP-MSKARQGAIINMSSVVGLMGNIGQANYAASKAGLIGFTKSVAREVAAR
31      NYEAISLLLPG-MVRARWGRIVLLSSVVAQSGMAGLSAYGAAKGALEALVKSLALEVGRR
                          *  *:   *  ..         *  .:*  :  :   :  :

11      GIDVVIIEPGGIK-TPWGLIAADHLE-----------E---SSRNGVYAAQAQRVAANMR
3       DIKVTTIQPGIVE-TPFSEVRFHGDA-----------ERAKSVYAGIDAIQPEDVADVVL
37      QIRVTNVEPGLCGGTEFSNIRFKGDD-----------ARAKKLYENVEYVSPQDIANIVL
4       GITVNAYAPGIVK-TPMMYDIAHEVGKNAGKDDEWGMQTFAKDITLKRLSEPEDVAAAVS
17      GVRVNAIAPGFIE-SDMTDAIPEKMK-----------DAMLAQVPMKRIGQAEEVAEVAA
31      AITVNAVAPGYIQ-TPMTQSLSPRQQ-----------ERYLQRTGAARPGTPQDVAGPVA
         : *    **    :                                     : :*

11      RLYSPSS--NLSEPKVISNAILRALEARRPKTRYLVGFGAKPSVFLHTVLPDRLFDKVAR
3       YVTNQPKRLQISDVTIMANQQAA-------------G-------FMV-------------
37      WLNQQPEHVNINRIEVMPTAQTF-------------A-------PLN----------VAR
4       FLAGPDSNYITGQTIIVDGGMQF----H--------------------------------
17      FLAG--QEYLTGQTIAIDGGMTM----Q--------------------------------
31      FLLSDDAAYVNGQILHVDGAMGV----GNP------------------------------
         :         .    :

11      RIF
3       ---
37      ---
4       ---
17      ---
31      ---
```

B. SDR percentage identity matrix for 3,4,11,17,31,37

**11**	100.00	33.05	31.38	26.18	27.04	25.43
**3**	33.05	100.00	39.18	27.39	28.75	21.21
**37**	31.38	39.18	100.00	24.79	21.94	23.38
**4**	26.18	27.39	24.79	100.00	31.80	28.88
**17**	27.04	28.75	21.94	31.80	100.00	31.47
**31**	25.43	21.21	23.38	28.88	31.47	100.00

Figure S2. Graph displaying activity with (*S*)-**18** and (*R*)-**18** with SDR-17 and SDR-31 at a range of pHs

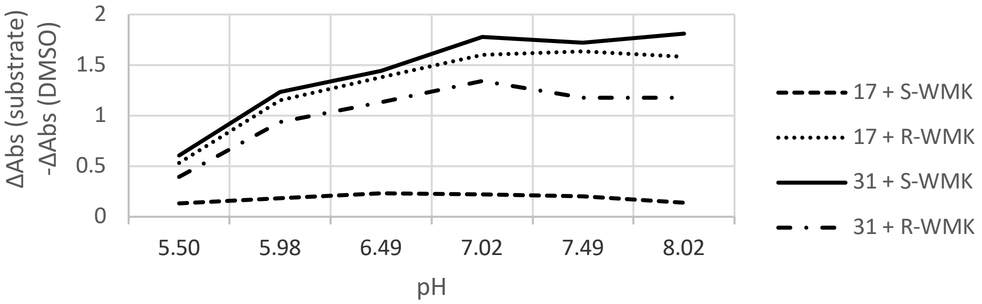

Reaction conditions (200 µL volume): **18** (5 mM), clarified cell lysate (0.4 mg/mL), NADPH (1 mM), KPi (100 mM), DMSO (10%, v/v). Reactions were shaken for 95 mins at 25 °C, performed in triplicate and quantified by the spectrophotometric assay at 340 nm.

Figure S3. SDS PAGE showing induced recombinant protein expression in *E.coli*.

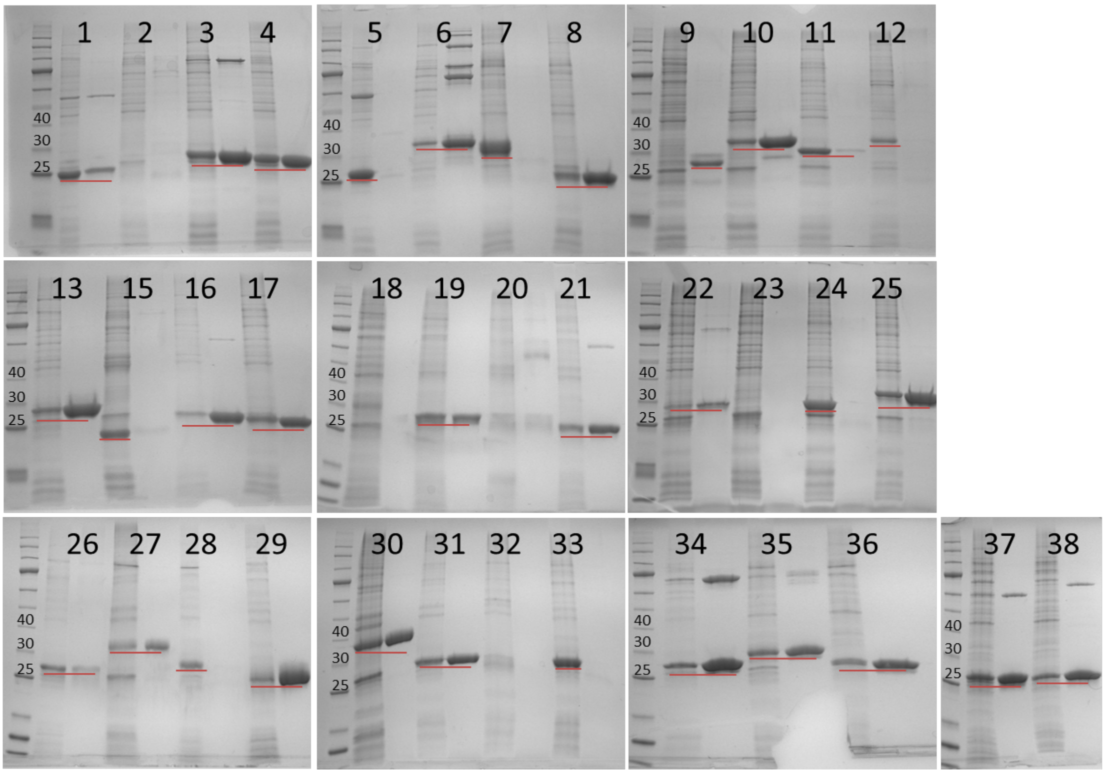

Numbers in the well line correspond to SDR number IDs. Each enzyme has two lanes, crude lysate and purified fraction from left to right. Bands corresponding to induced protein are underlined in red either in the crude lysate lane or both lanes in the case of successful purification. Markers are given in kDa (10-230 Broad range, NEB). Protein sizes in kDa calculated using the online tool expasy protparam as follows; 1) 29, 2) 27.5, 3) 29.5, 4) 29.4, 5) 30.2, 6) 30.7, 7) 29.8, 8) 28, 9) 28.9, 10) 31.5, 11) 33.2, 12) 35.6, 13) 29.6, 15) 22.9, 16) 28.4, 17) 28.5, 18) 33.3, 19) 31.2, 20) 30.1, 21) 29, 22) 34, 23) 29.7, 24) 31.5, 25) 34.3, 26) 29, 27) 33.1, 28) 29.1, 29) 29.2, 30) 32.6, 31) 27.4, 32) 30.1, 33) 28.96, 34) 29.2, 35) 30.7, 36) 28.5, 37) 30.2, 38) 28.5.

Figure S4. HPLC calibration curves for A. (*S*)-WMK, (*S*)-**18**. B. (*R*)-WMK (*R*)-**18**. C (4a*R*,5*R*)-**22**. D. (4a*R*,5*S*)-**22**. E. (4a*S*,5*S*)-**22.** Quantification by HPLC on a Chiralcel OJ column with 4% isopropanol/hexane mobile phase 0.5 mL/min flow rate and detection at 230 nm.

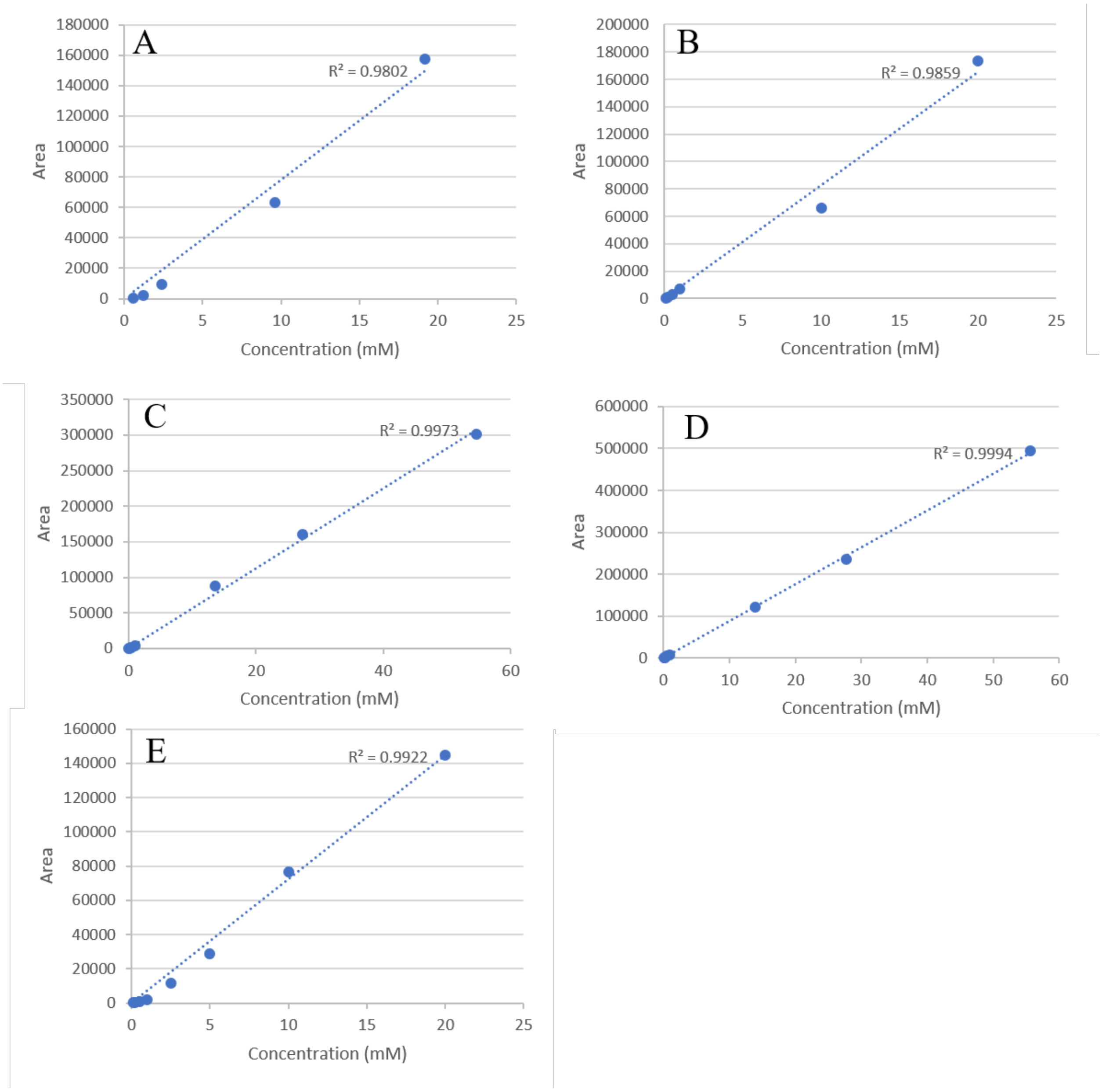

Figure S5. HPLC trace of the products of the reduction of *rac*-WMK-**18** using $NaBH_4$: (4a*R*,5*S*)-**22** Rt = 54.0 min, (4a*R*,5*R*)-**22** Rt = 61.2 min, (4a*S*,5*S*)-**22** Rt = 85.9 min. HPLC conditions were 4% iPrOH:hexane, 120 min, 0.5 mL/min at 230 nm, on a Chiracel OJ column.

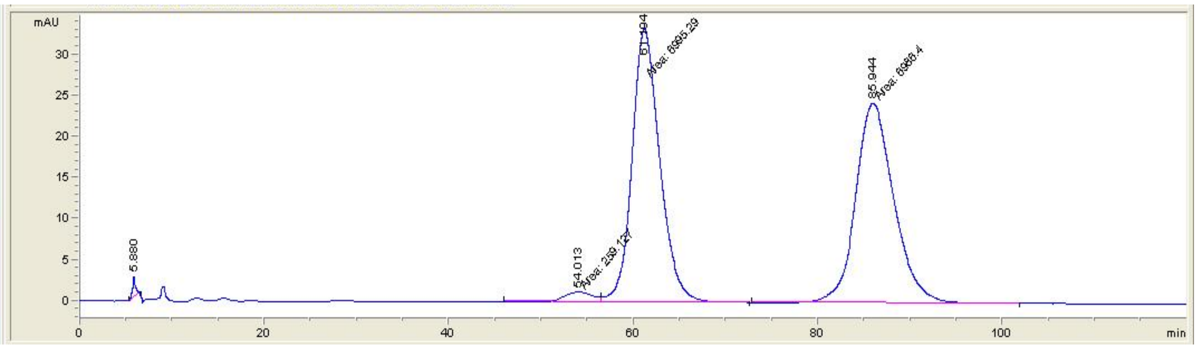

Figure S6. Characterisation data for (4a*R*,5*S*)-**22** from the scaled-up reaction using SDR-17**. A**. HPLC trace of (4a*R*,5*S*)-**22** starting from (*R*)-**18** using SDR-17. Rt = 54 min. HPLC using Chiralcel OJ column with 4% isopropanol/hexane mobile phase 0.5 mL/min flow rate and detection at 230 nm. **B**. ^{1}H and ^{13}C NMR spectra of (4a*R*,5*S*)-**22**.

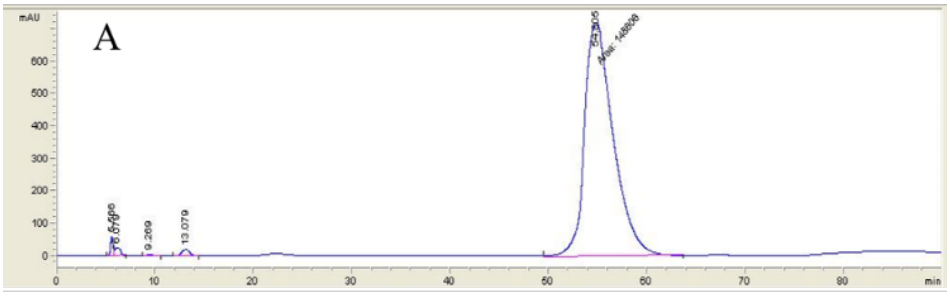

B

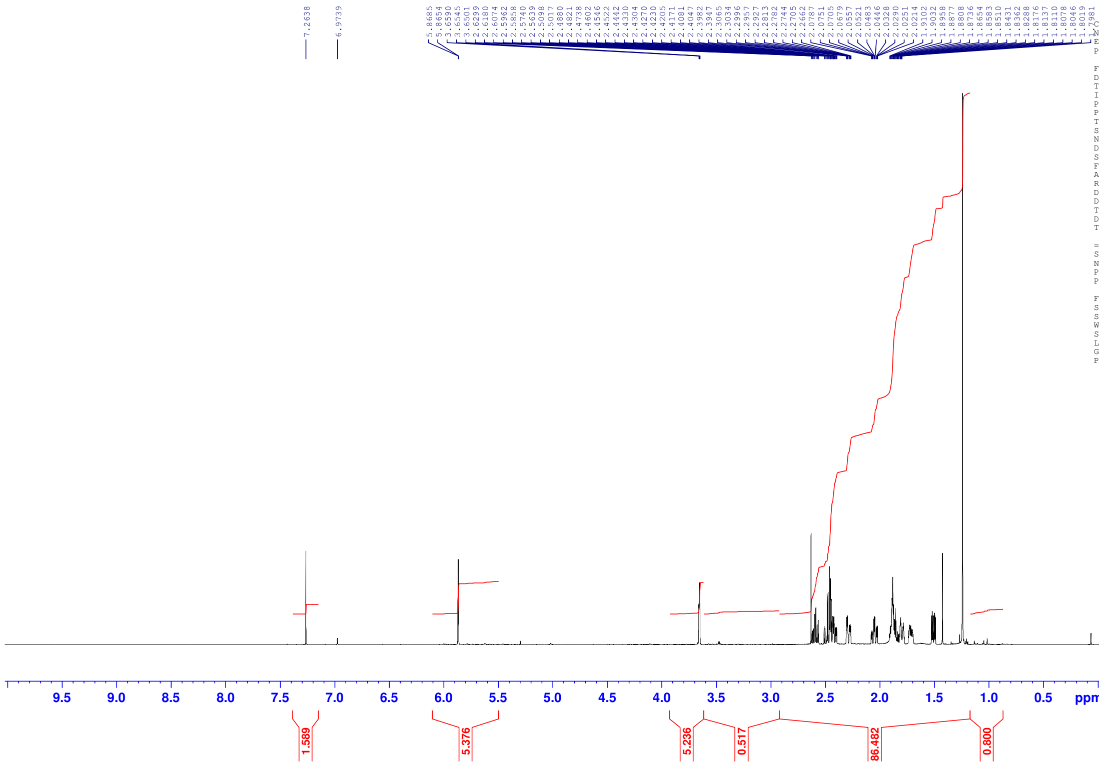
7.2638
6.9739
9.5
9.0
8.5
8.0
7.5
7.0
6.5
6.0
5.5
5.0
4.5
4.0
3.5
3.0
2.5
2.0
1.5
1.0
0.5
ppm
1.589
5.376
5.236
0.517
86.482
0.800

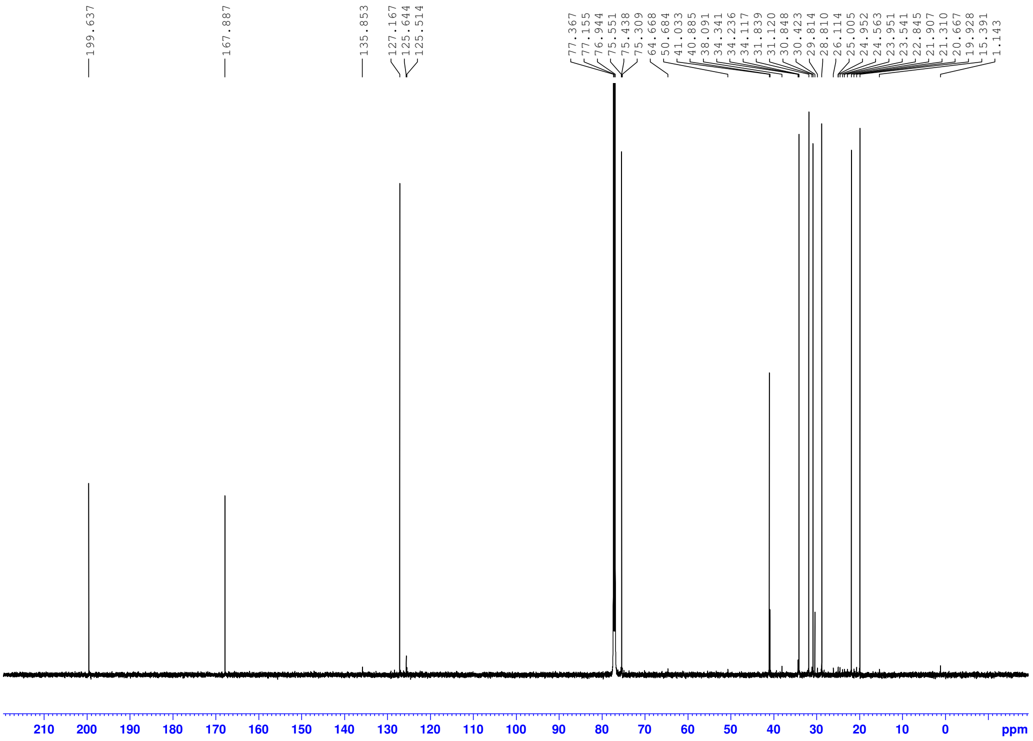
199.637
167.887
135.853
127.167
125.644
125.514
77.367
77.155
76.944
75.551
75.438
75.309
64.668
50.684
41.033
40.885
38.091
34.341
34.236
34.117
31.839
31.120
30.848
30.423
29.814
28.810
26.114
25.005
24.952
24.563
23.951
23.541
22.845
21.907
21.310
20.667
19.928
15.391
1.143
210
200
190
180
170
160
150
140
130
120
110
100
90
80
70
60
50
40
30
20
10
0
ppm

Figure S7. ^{1}H and ^{13}C NMR spectra of (4a*R*,5*R*)-**22**.

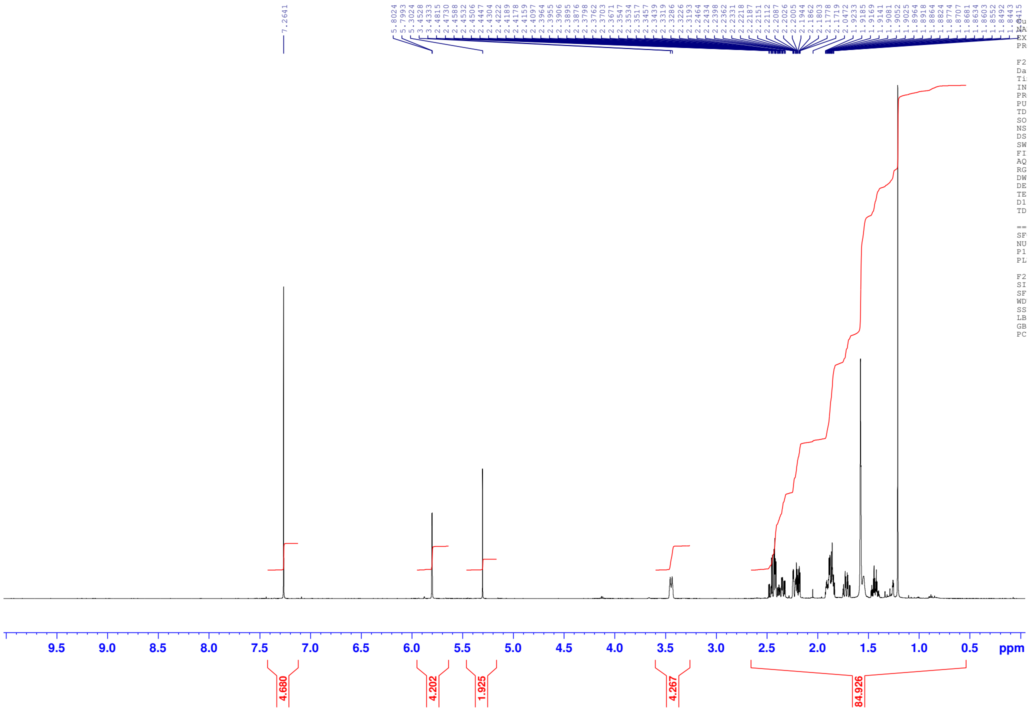

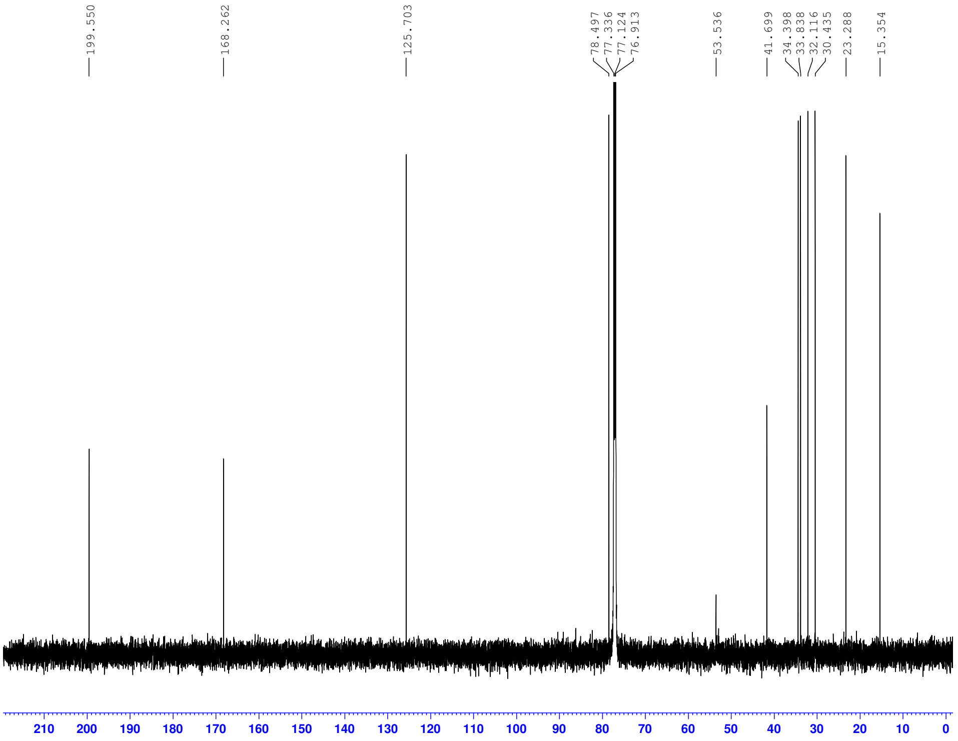

Figure S8. ^{1}H and ^{13}C NMR spectra of (4a*S*,5*S*)-**22**.

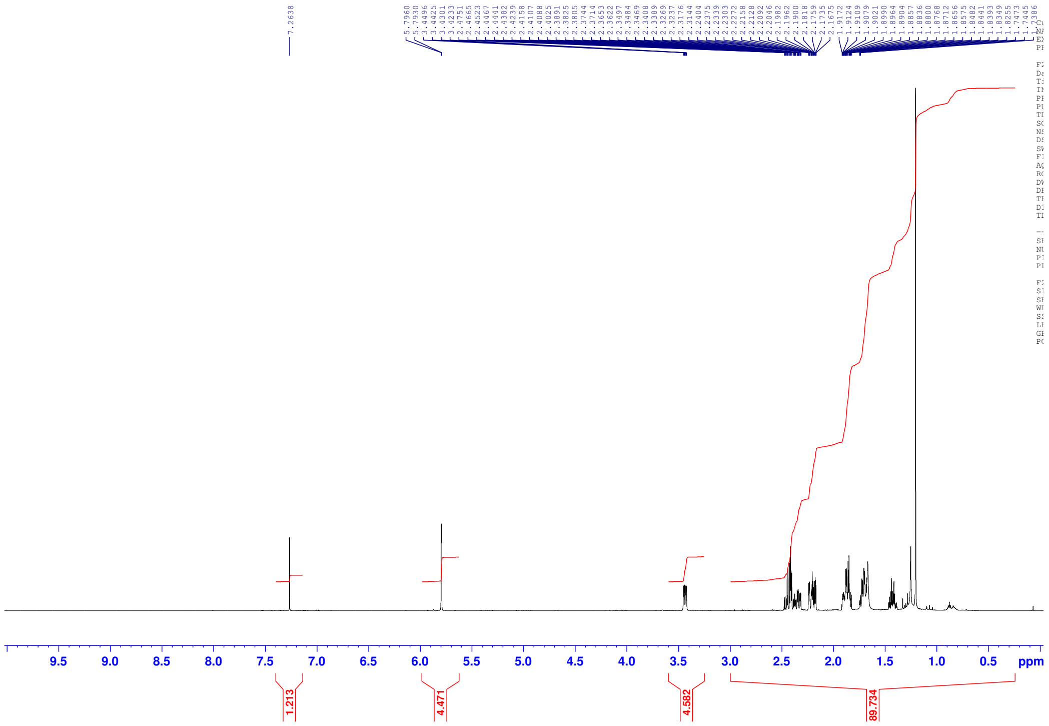

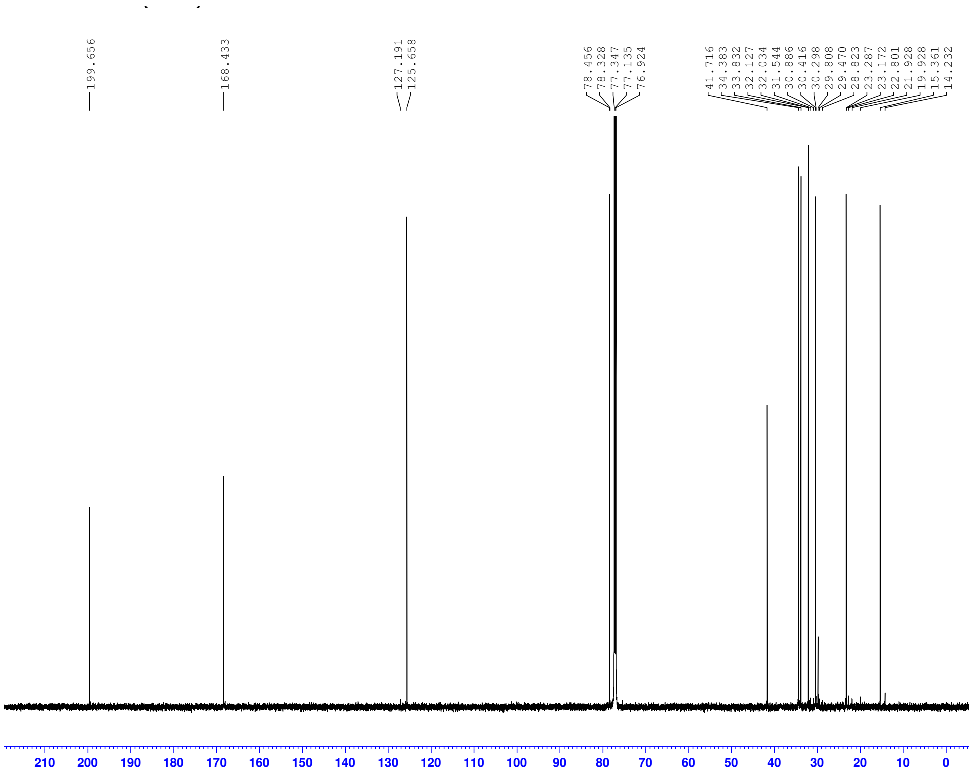

Supplement: Supplementary file 1 — Supplementary [file ADSC-363-3044-s001.pdf]
